# Obesity-induced mesenteric PVAT remodelling is sexually dimorphic, but not driven by ovarian hormones: Short title: Obesity induces sex-specific responses in mesenteric PVAT

**DOI:** 10.1186/s12933-025-02596-w

**Published:** 2025-01-24

**Authors:** Lisa Ivatt, Mhairi Paul, Allende Miguelez-Crespo, Patrick W. F. Hadoke, Matthew A. Bailey, Ruth A. Morgan, Mark Nixon

**Affiliations:** 1 Centre for Cardiovascular Science, University of Edinburgh, Edinburgh, Scotland, UK; 2 Scotland’s Rural College, Edinburgh, Scotland UK

**Keywords:** Obesity, Perivascular adipose tissue, Vascular dysfunction, Insulin resistance, Sex-specific response

## Abstract

**Background:**

Obesity, a major risk factor for cardiovascular disease (CVD), is associated with hypertension and vascular dysfunction. Perivascular adipose tissue (PVAT), a metabolically active tissue surrounding blood vessels, plays a key role in regulating vascular tone. In obesity, PVAT becomes dysregulated which may contribute to vascular dysfunction; how sex impacts the remodelling of PVAT and thus the altered vascular contractility during obesity is unclear.

**Objective:**

To investigate sex-specific PVAT dysregulation in the setting of obesity as a potential driver of sex differences in vascular pathologies and CVD risk.

**Methods:**

Adult male and female C57Bl/6J mice were fed an obesogenic high-fat diet (HFD) or regular chow for 16 weeks. Mesenteric PVAT (mPVAT) was isolated for RNA-sequencing and histological analysis, and mesenteric arteries were isolated for assessment of vascular function by wire myography. In a separate study, female mice were subjected to bilateral ovariectomy prior to dietary intervention to determine the contribution of ovarian hormones to PVAT dysregulation.

**Results:**

Transcriptomic analysis of mPVAT revealed sexually dimorphic responses to HFD, with upregulation of extracellular matrix (ECM) remodelling pathways in male but not female mice. Histological and RT-qPCR approaches demonstrated increased collagen deposition and ECM remodelling in mPVAT from obese male compared with obese female mice. Assessment of vascular function in mesenteric arteries -/+ PVAT revealed that in obesity, mPVAT impaired endothelium-mediated vasodilation in male but not female mice. Ovariectomy of female mice prior to HFD administration did not alter ECM transcript expression or collagen deposition in mPVAT compared to sham-operated female mice.

**Conclusions:**

Obesity induces sex-specific molecular remodelling in mPVAT, with male mice exhibiting unique upregulation of ECM pathways and increased collagen deposition compared to females. Moreover, the relative protection of female mice from obesity-induced mPVAT dysregulation is not mediated by ovarian hormones. These data highlight a potential sex-specific mechanistic link between mPVAT and mesenteric artery dysfunction in obesity, and provides crucial insights for future development of treatment strategies that consider the unique cardiovascular risks in men and women.

**Graphical abstract:**

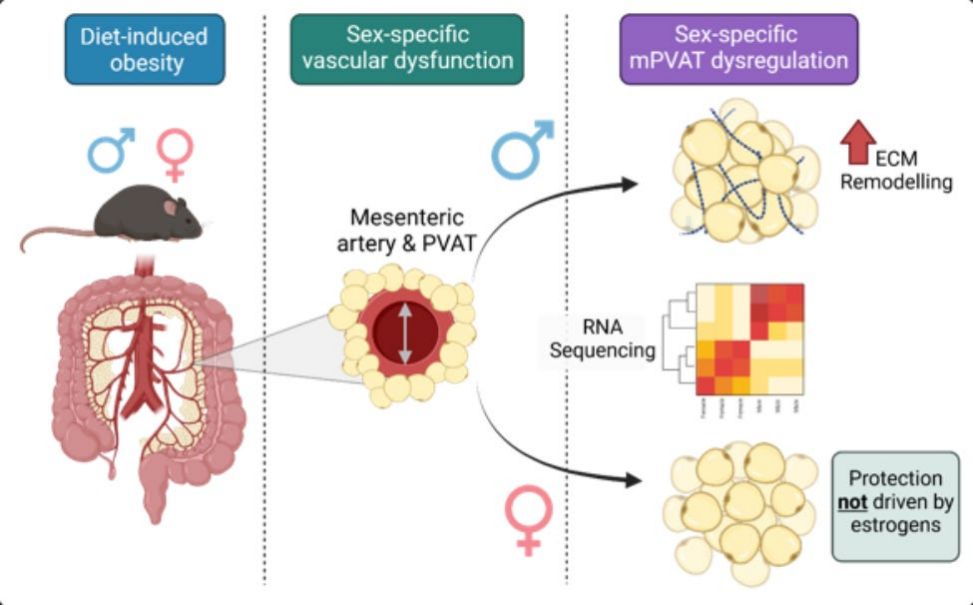

**Supplementary Information:**

The online version contains supplementary material available at 10.1186/s12933-025-02596-w.

## Introduction

Obesity, characterised by excessive fat accumulation, is an escalating problem and a primary risk factor associated with cardiovascular disease (CVD) [1]. Hypertension is a comorbid condition frequently associated with obesity, with the Framingham Heart Study showing that 78% of cases of essential hypertension in men and 65% in women could be attributed to excess body fat [2]. In line with this, even a modest reduction in weight can decrease blood pressure in hypertensive patients [3]. Notably, sex differences play a significant role in CVD risk, exemplified in women who experience a sharp increase in hypertension and CVD prevalence after menopause [4, 5]. While the precise mechanisms driving obesity-related vascular dysfunction and the underlying sexual dimorphism are not fully understood, several potential pathways, including the interaction of adipose tissue with the vasculature, may be involved.

Adipose tissue, originally thought to only be a form of energy storage, is now recognised as a highly active, endocrine tissue [6]. Specific fat depots have been implicated in the pathogenesis of CVD, including perivascular adipose tissue (PVAT) that surrounds peripheral blood vessels [7, 8]. PVAT is a dynamic and metabolically active tissue which contributes to the regulation of vascular tone [8, 9, 10]. In health, PVAT exerts anti-contractile effects on the vasculature in response to different vasoconstrictors, modulating vascular resistance and protecting against excessive increases in blood pressure [11, 12]. However, in obesity this anti-contractile effect is diminished, an effect hypothesised to contribute to increased blood pressure [12, 13]. The underlying mechanisms driving obesity-associated vascular dysfunction remain unclear. Investigation into this is complicated by two major factors: the presence of distinct anatomical PVAT depots, with conflicting reports on their role in vascular regulation, and the presence of sex-specific responses to obesity-induced PVAT and vascular dysfunction.

In the thoracic aorta, which is a large conduit artery, PVAT (termed tPVAT) has a brown adipose tissue (BAT)-like phenotype [14, 15, 16], with demonstrated sex differences in both PVAT itself and PVAT interactions with the artery during obesity [17, 18]. However, the influence of sex on PVAT surrounding resistance arteries such as those in the mesentery, in response to obesity, is less clear. Clinically, this is an important relationship as resistance arteries strongly influence blood pressure and dysregulation contributes to hypertension [19]. Moreover, mesenteric PVAT (mPVAT) is more WAT-like and forms part of the visceral WAT that is strongly linked with CVD [15, 16]. Consequently, the role of mPVAT has become a significant area of interest in elucidating the mechanisms underlying obesity-induced vascular dysfunction. Here we utilised a transcriptomics approach to provide the first parallel characterisation of mPVAT and tPVAT in a mouse model of obesity-induced vascular dysfunction. Subsequently, we demonstrate the anatomical heterogeneity of PVAT by determining that tPVAT responds less dynamically to diet-induced obesity than mPVAT, with limited sexual dimorphism. In contrast, we reveal that in mPVAT, male mice undergo significant ECM remodelling in response to HFD compared to female mice. Moreover, in mPVAT, we show that the remodelling exhibited in obese male mice associates with endothelium-dependent impaired vasodilation in mesenteric arteries. Finally,we establish that the relative protection of females from obesity-induced mPVAT dysfunction is not mediated by ovarian hormones. These findings provide crucial insights into the role of specific PVAT depots in the response to obesity, and shed further light on the links between PVAT and vascular dysfunction.

## Methods

### Animals

Studies in mice were designed in compliance with all relevant ethical regulations under project licences granted by the UK Home Office, and were approved by the University of Edinburgh Animal Welfare and Ethical Review Board and Directive 2010/63/EU of the European Parliament on the protection of animals used for scientific purposes.

Male and female C57Bl/6J mice were purchased from Harlan, UK between 8 and 10 weeks old. Mice were housed in groups of 4–5 mice per cage unless otherwise stated, at 21 °C on a 12 h light/dark cycle (0700–1900 h) with free access to water and food, as indicated. Mice were randomised to receive either regular chow diet (2.5% kcal fat; Special Diet Services 801002;) or a high-fat diet (58% kcal fat with sucrose; Research Diets D12331) for up to 16 weeks. A cohort of female mice underwent ovariectomy (OVX) under inhalation anaesthesia using isoflurane. Sham-operated mice were subjected to similar surgery, with ovaries exposed but not removed. Mice were provided with analgesia (oral self-administration carprofen 25 mg/kg in drinking water) over the course of the next 7 days while they recovered, prior to administration of high-fat diet. Animals were allowed to recover for 7 days before starting high-fat diet. Mice were culled between 0800 h and 1000 h by decapitation to minimise the stress response (< 1 min between handling and decapitation). Trunk blood was collected and allowed to clot, before being subjected to centrifugation (1,500 g, 10 min) to obtain serum. The mesenteric bed was isolated and segments (1 cm) of second order arteries with surrounding PVAT (mPVAT) were dissected out for analysis. In some studies, the PVAT from the thoracic aorta (tPVAT) was also isolated and dissected out for further analysis.

### Metabolic measurements

At 15 weeks dietary intervention, glucose tolerance tests (GTT) were performed on mice fasted for 6 h. Animals received an intraperitoneal (IP) injection of glucose (2 g/kg of body weight). Blood glucose levels were determined via tail venesection at various time points post-injection (15, 30, 45, 90 min) using a point-of-care glucometer (Accu-Chek; Roche). Blood was collected into EDTA-coated microvette tubes (Sarstedt; #18.1321) from fasted mice to determine fasting insulin levels. Plasma was obtained following centrifugation (10,000 g, 5 min), and samples assessed using a commercial insulin ELISA (Millipore; #EZRMI-13 K) following manufacturer’s instructions. Homeostatic model assessment of insulin resistance (HOMA-IR) was calculated by using the formula (Fasting insulin (mg/dL) × Fasting glucose (mmol/L)/22.5. At 16 weeks, blood pressure was measured using non-invasive tail cuff plethysmography (CODA 8; Kent Scientific) as previously described [20]. Blood pressure measurement experiments were conducted in a designated quiet room, where mice acclimatised for a 1-hour period before experiments began. Each recording session consisted of 15 to 25 inflation and deflation cycles per set, of which the first 5 cycles were “acclimation” cycles and were not used in the analysis. Mice were habituated for at least 3 consecutive days before experimental blood pressure measurements were recorded to reduce stress-induced responses. Blood pressure was then recorded on 3 consecutive days. Serum 17β-estradiol was quantified at cull using a commercial ELISA (Abcam; #ab108667) following manufacturer’s instructions.

### Extraction and quantification of mRNA by RT-qPCR

Total RNA was extracted from adipose using a RNeasy Mini kit (Qiagen Inc., Valencia, CA, USA; #74106) according to the manufacturer’s instructions. The tissue was mechanically disrupted in either QIAzol (Qiagen; #79306). cDNA was synthesised using a QuantiTect Reverse Transcription kit (Qiagen; #205311) according to the manufacturer’s instructions. A quantitative real-time polymerase chain reaction was performed using a LightCycler 480 (Roche Applied Science, Indianapolis, IN, USA). The qRT-PCR was performed using SYBR Green (iTaq, Bio-Rad; #172–5151). Primers were designed using sequences from the National Centre of Biotechnological Information and primer sequences are included in Supplementary Table 1. Samples were analysed in triplicate and amplification curves plotted (y axis, fluorescence; x axis, cycle number). Triplicates were deemed acceptable if the standard deviation of the crossing point was < 0.5 cycles. All primers were previously calibrated and used at efficiencies between 90 and 110%. Data analysis was performed using the Pfaffl method [21], with the abundance of each gene expressed relative to the mean of three housekeeping genes (*Hprt*,*  18S*,* Tbp)*. Paucity of sample in some studies mean that samples were excluded due to insufficient RNA quality for downstream analysis. N numbers are indicated in individual figure legends.

### Bulk RNA sequencing analysis

Samples (*n* = 5 per experimental group) of mPVAT and tPVAT were processed for RNA isolation (as above). Total RNA was quantified using a Nanodrop spectrophotometer (Thermo Scientific), and integrity was assessed using an Agilent 2100 Bioanalyzer (Agilent Technologies Inc.) and the Agilent RNA 6000 Nano kit (Agilent; #5067 − 1511). Library preparation and transcriptome sequencing were conducted by Novogene Co. Ltd. cDNA libraries were sequenced using the Illumina NovaSeq platform (Illumina Inc.). Bioinformatic analysis was performed by FIOS Genomics (Edinburgh, UK). The quality of the data was assessed by the FastQC control tool. Reads were aligned to a mouse reference genome build GRCm39 using the STAR aligner, followed by calculation of alignment and mapping statistics. At least 83.5% (mPVAT) and 82.6% (tPVAT) of read pairs were uniquely mapped to one region of the genome. Analysis was performed using log2 fold of change (FC), calculated individually for each comparison; for example, between male and female mice under chow diet, or between chow and HFD in male mice. Volcano plots for single comparisons are presented as adjusted P-values. Further analyses were performed correcting for multiple testing (FDR adjusted) with a significance threshold of *P* < 0.05.

### Histological analysis

Adipose tissue samples were fixed overnight in 4% paraformaldehyde, before embedding in paraffin. Sections (5 μm) were placed onto slides (SuperFrost, Fisher; #1255015) and stained with hematoxylin and eosin (H&E) or picrosirius red (PSR) and images captured using an Nikon Eclipse Ci-L Plus microscope (Nikon). Quantification of adipocyte area was performed using FIJI software (ImageJ) with Adiposoft plugin. Paucity of sample meant not all mice had histological analysis performed. N numbers are indicated in individual figure legends.

Ex vivo*wire myography*.

Animals were culled between 0800 and 1000 h for artery collection. ~ 2 mm segments of second order mesenteric artery (< 240 μm diameter) with PVAT either intact or removed were mounted on two stainless steel intraluminal wires (diameter 40 μm) in a multi-myography system (Danish Myo Technology (DMT), AD Instruments, #610 M) containing physiological salt solution (PSS; 119.0 mM NaCl, 4.7 mM KCl, 2.5 mM CaCl_2_, 1.2 mM MgSO_4_, 25.0 mM NaHCO_3_, 1.2 mM KH_2_PO_4_, 27.0 µM EDTA, 5.5 mM d-glucose) aerated at 37 °C with 95% O_2_/5% CO_2_. Following equilibration (30 min), each artery was subjected to a standard normalisation procedure (using the DMT Normalisation Module on LabChart; AD Instruments) and then set at 0.9 L100 as its optimum resting setting. In all studies, the viability of each artery was first confirmed by a contractile response of a high-potassium physiological salt solution (KPSS; 125 mM KCl, 2.5 mM CaCl_2_, 1.2 mM MgSO_4_, 25.0 mM NaHCO_3_, 1.2 mM KH_2_PO_4_, 27.0 µM EDTA, 5.5 mM d-glucose), repeated three times using the DMT Normalisation Module on LabChart (AD Instruments). Alpha-adrenoceptor-mediated vasoconstriction was assessed by exposing vessels to cumulative concentrations (1 nmol/L– 3 µmol/L) of phenylephrine (PE). To assess vasodilator capacity, vessels were preconstricted with half-maximal effective concentration (EC_50_) of PE, and cumulative concentration curves (1 nmol/L–3 µmol/L) were obtained to acetylcholine (ACh) and sodium nitroprusside (SNP). A 30-min washout was allowed between concentration-response curves. For vasoconstriction, the maximum contraction values (in mN) induced by each PE concentration were normalised to the maximum contraction induced by KPSS at the start (%KPSS). For vasodilation, the minimum tension values (in mN) measured for each ACh or SNP concentration were normalised to the initial contraction force induced by PE-preconstriction before the start of vasodilation drugs application (% of PE preconstriction). Vessel death, determined by lack of response to KPSS, occurred in some instance meaning not all mice had myography analysis. N numbers are indicated in individual figure legends.

### Statistical analysis

Data are presented throughout the paper as mean ± SD. Prior to statistical testing, all data was assessed for normal distribution using the Shapiro-Wilk normality test. Comparisons between two means were by unpaired t-test. Comparisons of measurements across two factors (sex and diet) were performed by two-way ANOVA, followed by a post hoc test (Tukey). Comparisons of measurements over time/concentration were performed by two-way ANOVA with repeated measures, followed by a post hoc test (Sidak). All statistical analyses were carried out using GraphPad Prism V9. Differences were deemed statistically significant when *P* < 0.05.

## Results

### HFD induces preferential weight gain and glucose intolerance in male mice vs. female mice

**Fig. 1:**
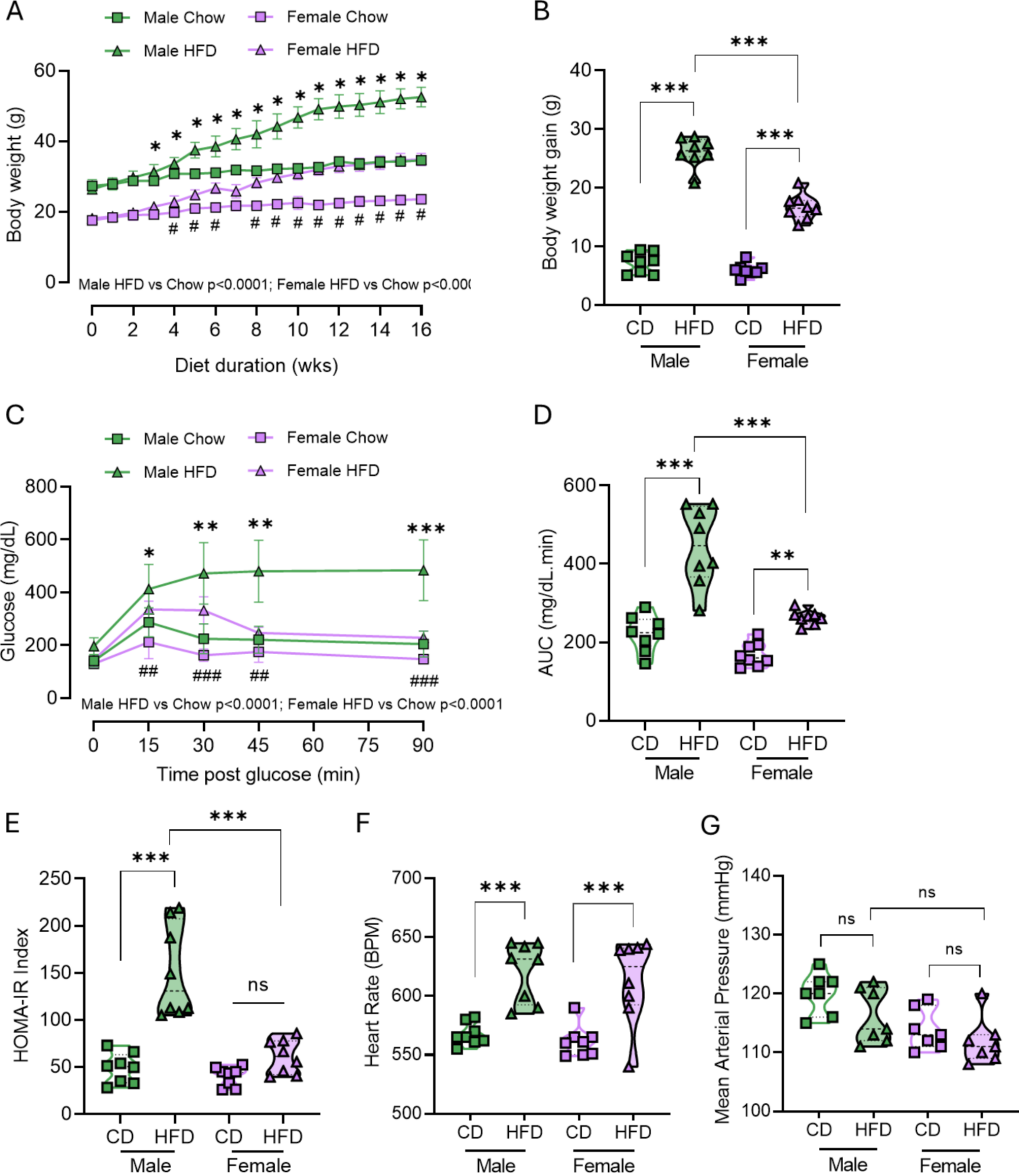
*Male mice are more sensitive to HFD-induced weight gain and glucose intolerance* Adult 8-week old male (green) and female (purple) C57Bl/6J mice were fed either a high-fat diet (HFD, triangle) or Control chow diet (CD, square) for 16 weeks. Body weights were measured weekly (**A**). Weight gain (g) at 16 weeks was calculated from start of diet (**B**). Blood glucose levels during an IP glucose tolerance test performed at 15 weeks of diet; glucose was measured in plasma samples during a glucose tolerance test (**C**) and corresponding area under the curve (AUC) (**D**). HOMA-IR measure of insulin sensitivity was determined at 15 weeks of diet (**E**). Heart rate (**F**) and mean arterial pressure (**G**) were measured by tail-cuff plethysmography at 16 weeks of diet. Data are mean ± SD (*N* = 8 per group). **A**, **C** analysed by RM 2-way ANOVA. **B, D, E-G**,,analysed by 2-way ANOVA with post-hoc test. **P < 0.01, ***P < 0.001, ***P < 0.0001

Administration of 16-week HFD resulted in weight gain (Fig. 1A), which was greater in male mice (Fig. 1B). HFD also induced glucose intolerance in both sexes compared with their chow-fed controls. This glucose dysregulation was also greater in male, compared with obese female, mice (Fig. 1C-D). HOMA-IR was increased by HFD in male, but not female mice (Fig. 1E). In contrast, HFD resulted in similar increases in heart rate between the sexes compared to chow-fed controls (Fig. 1F). However, no increase in mean arterial pressure was observed in HFD-fed mice, in either male or female mice (Fig. 1G).

### PVAT exhibits depot- and sex-specific response to HFD

**Fig. 2:**
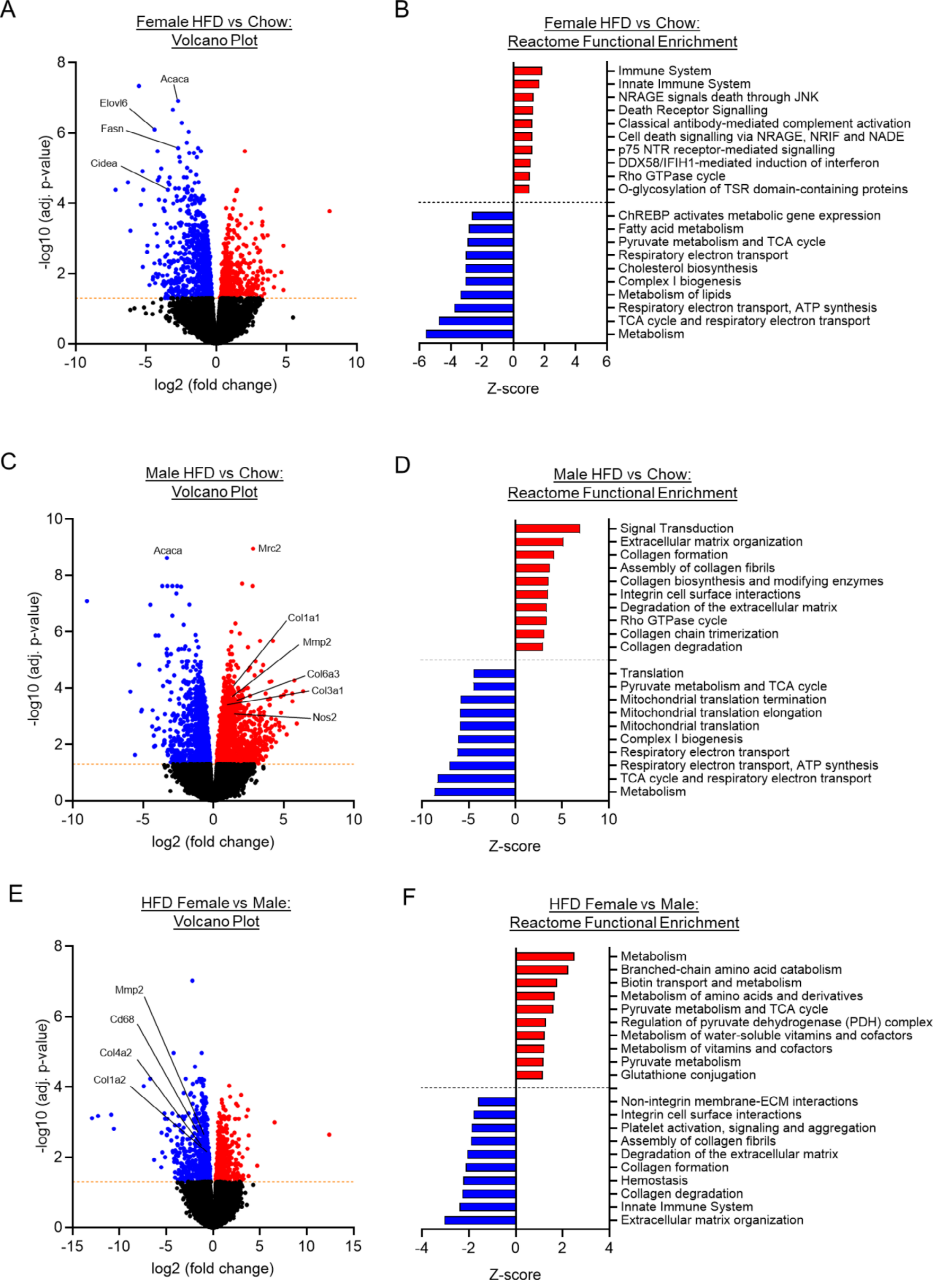
*Mesenteric PVAT (mPVAT) displays a sex-specific transcriptional response to high fat diet (HFD)* Volcano plots showing the number of significantly (adj. p-value < 0.05) upregulated (red) and downregulated (blue) differentially expressed genes (DEGs) in mPVAT from HFD vs. Chow Female (**A**), HFD vs. Chow Male (**C**), and Female vs. Male HFD (**E**). DEGs from each comparison were included in a Reactome pathway functional enrichment analysis. The top significantly enriched upregulated (red) and downregulated (blue) Reactome pathways are represented by Z-score for HFD vs. Chow Female (**B**), HFD vs. Chow Male (**D**), and Female vs. Male HFD (**F**). Analyses were performed in five animals (*N* = 5) per experimental group

**Fig. 3:**
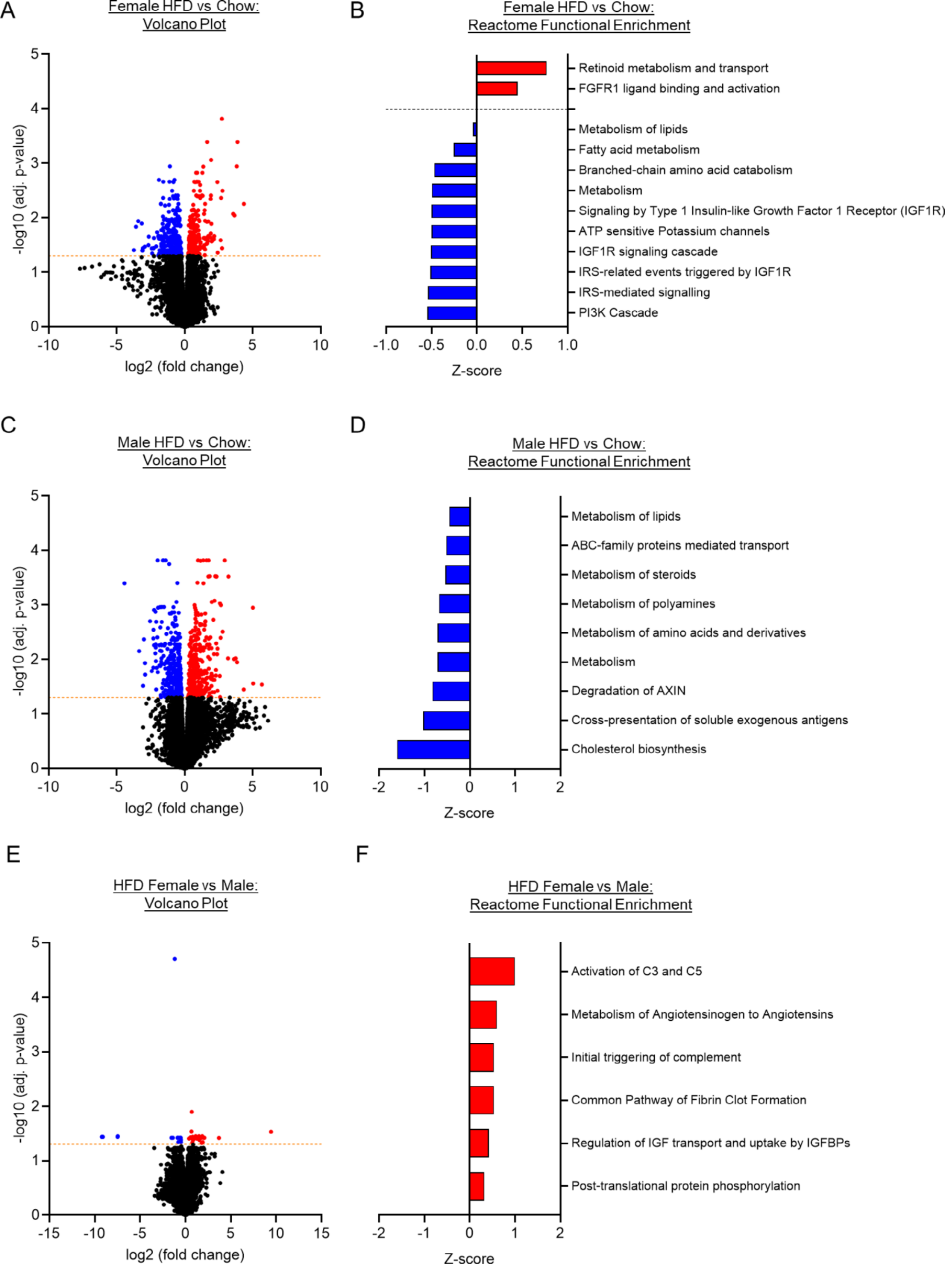
*The transcriptomic response of thoracic PVAT (tPVAT) to high fat diet (HFD) is similar in male and female mice* Volcano plots showing the number of significantly (adj. p-value < 0.05) upregulated (red) and downregulated (blue) differentially expressed genes (DEGs) in tPVAT from HFD vs. Chow Female (**A**), HFD vs. Chow Male (**C**), and Female vs. Male HFD (**E**). DEGs from each comparison were included in a Reactome pathway functional enrichment analysis. The top significantly enriched upregulated (red) and downregulated (blue) Reactome pathways are represented by Z-score for HFD vs. Chow Female (**B**), HFD vs. Chow Male (**D**), and Female vs. Male HFD (**F**). Analyses were performed in five animals (*N* = 5) per experimental group

We performed bulk RNA sequencing (RNA-seq) on both mesenteric PVAT (mPVAT) (Fig. 2) and thoracic PVAT (tPVAT) (Fig. 3) from male and female mice under chow-fed and HFD-fed conditions. For each depot, we compared the effect of diet and of sex on the molecular signature. In mPVAT, following adjustment for multiple comparison testing, no significant differentially expressed genes (DEGs, adjusted P-value < 0.05) were observed between male and female mice under chow-fed conditions (Supplementary Table 2). In response to HFD, female mice exhibited > 1400 DEGs (Fig. 2A), with almost twice as many being downregulated vs. upregulated (911 vs. 582). Functional enrichment analysis was used to identify key biological pathways amongst these DEGs, highlighting significant enrichment of several Reactome pathways encompassing downregulated DEGs relating to metabolism in HFD females (Fig. 2B; Supplementary Table 3). In male mice, the response to HFD was greater than in female mice, with more than twice as many DEGs vs. females (3474 vs. 1493). In contrast to female mice, male mice exhibited a greater proportion of upregulated DEGS compared to those downregulated (1925 vs. 1549), including a number of collagens (*Col1a1*,* Col3a1*,* Col6a3*) (Fig. 2C). This unique response to HFD was further evidenced following functional enrichment analysis in which upregulated DEGs mapping to multiple pathways relating to extracellular matrix remodelling were identified under HFD conditions (Fig. 2D; Supplementary Table 4). Comparison of female vs. male mPVAT under HFD conditions also revealed > 1000 DEGs (Fig. 2E), indicating reduced expression of a number of collagens (*Col1a2*,* Col4a2*) in female mPVAT. Functional enrichment analysis provided further support for sex-specific differences in extracellular matrix remodelling, with numerous Reactome pathways relating to the extracellular matrix identified (Fig. 2F; Supplementary Table 5).

In tPVAT, HFD also induced a significant transcriptional response, albeit of a smaller magnitude than that observed in mPVAT and with reduced sexual dimorphism (Fig. 3). In female mice, 590 DEGs were identified in response to HFD, evenly split between upregulation and downregulation (284 vs. 306) (Supplementary Table 6). Functional enrichment analysis revealed that metabolism-related pathways were the most significantly enriched from the downregulated DEGs (Fig. 3B). In males, 773 DEGs were identified in response to HFD, with an equal split between upregulation and downregulation (393 vs. 380) (Fig. 3C). Significantly enriched Reactome pathways were only identified for DEGs that were reduced in response to HFD, and included metabolism-related pathways (Fig. 3D). Only a limited number of DEGs (56) were identified comparing HFD response in female vs. male mice (Fig. 3E). Functional enrichment analysis identified only 6 significantly enriched Reactome pathways, all encompassing upregulated DEGS and mapping to the complement cascade and regulation of IGF transport (Fig. 3F).

### Obesity preferentially induces collagen deposition and ECM remodelling in mesenteric PVAT from male vs. female mice

**Fig. 4:**
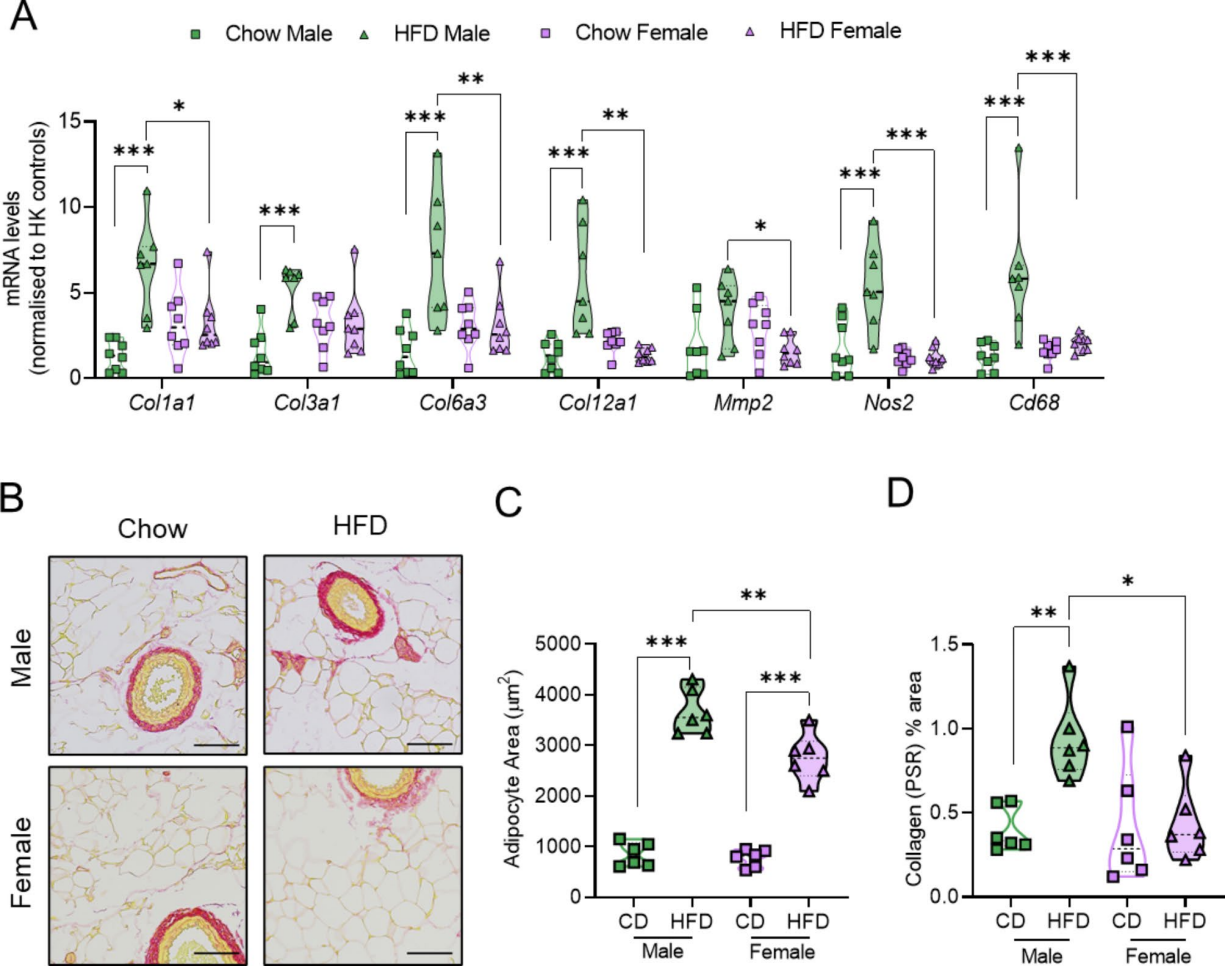
*High fat diet (HFD) preferentially induces adipocyte hypertrophy and adventitial collagen deposition in mesenteric PVAT (mPVAT) from male vs. female mice* Transcript mRNA levels of extracellular matrix (ECM) remodelling and inflammatory genes were determined by real-time PCR (**A**). Representative Picrosirius Red (PSR) images of sections from mPVAT (**B**). Scale bar = 80 μm. Analysis of adipocyte size (**C**) and percent (%) PSR staining (**D**). Data are expressed as mean ± SD (*n* = 6–10 per group), analysed by 2-way ANOVA with Tukey’s multiple comparisons post-hoc test. *P < 0.05, **P < 0.01, ***P < 0.001

Our RNA-seq data indicated that mPVAT exhibits a heightened sensitivity to diet and sex, making it an ideal target tissue to explore the mechanisms underlying vascular responses in obesity. To validate our transcriptomic results, we quantified mRNA transcripts of ECM remodelling and inflammatory genes identified from RNA-seq analysis in mPVAT (Fig. 4A). mRNA expression of *Col1a1*,* Col3a1*,* Col6a3*,* Col12a1*,* Nos2* and *Cd68* transcripts were increased in mPVAT from obese males compared to chow controls. In contrast, HFD did not induce expression of these transcripts in female mice. Significant differences were also observed in expression of *Col1a1*,* Col6a3*,* Col12a1*,* Mmp2*,* Nos 2* and *Cd68* transcripts between HFD female and HFD male mice. To determine whether transcriptional changes in collagen related genes resulted in altered mPVAT morphology, we performed histological analyses. HFD increased adipocyte area in the mPVAT of both male and female mice compared with chow-fed controls (Fig. 4B-C). However, under HFD conditions, mPVAT adipocytes from male mice were larger than those from female mice (Fig. 4C). Supporting our RNA-seq and qPCR data, collagen content, assessed by picrosirius red staining, was significantly increased in mPVAT from obese male mice, but not obese female mice, compared to chow-fed controls (Fig. 4D).

### Mesenteric PVAT reduces sensitivity of mesenteric arteries to adrenoceptor-mediated vasoconstriction in chow-fed male and female mice

**Fig. 5:**
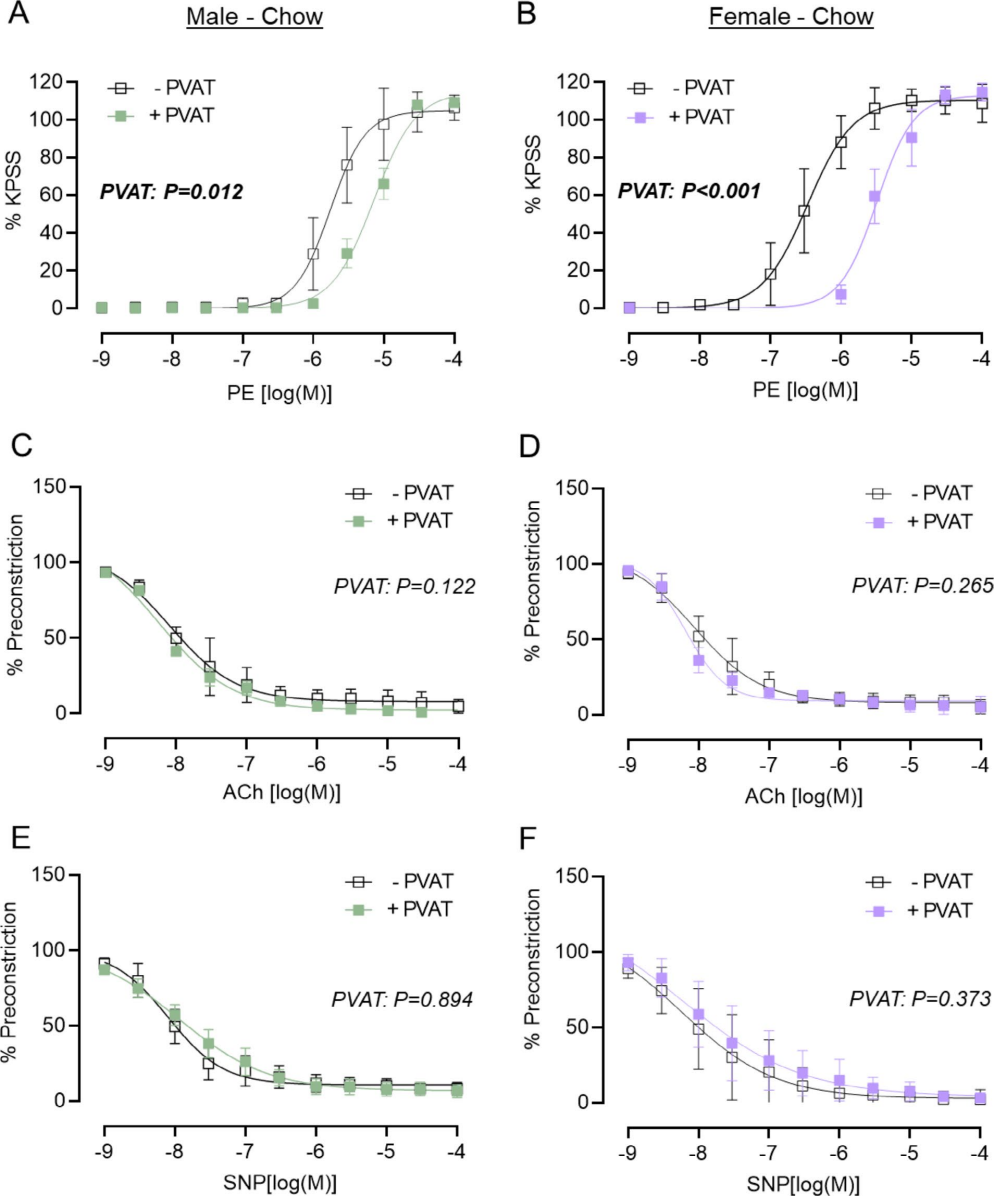
In chow-fed mice, PVAT reduced sensitivity of mesenteric arteries to PE-induced contraction in both sexes. Second order mesenteric arteries were dissected from male (left-hand panels **A**, **C**, **E**; green) and female (right-hand panels **B**, **D**, **F**; purple) C57Bl/6J mice after 16 weeks of chow diet. PVAT was either removed (-PVAT; white symbol) or left intact (+ PVAT; colour symbol) and vessels were mounted onto a wire myograph. Concentration response curves to phenylephrine (PE) (**A**, **B**), acetylcholine (ACh) (**C**, **D**) and sodium nitroprusside (SNP) (**E**, **F**) were obtained. For PE, contraction was normalised to the maximum response induced by a high potassium physiological salt solution (KPSS). For ACh and SNP, relaxation was normalised to a percentage of each vessel’s response to a half maximal concentration of PE (% Preconstriction). All concentration-response curves were generated by non-linear regression. LogEC 50 /IC 50 and %Emax/ %Preconstriction were calculated for each concentration-response curve (Supplementary Table 7). Data are group mean ± SD ( N = 6–8 per group), analysed by repeated measures two-way ANOVA. P < 0.05 was considered significant.

To first establish the physiological effect of mPVAT on vessel responses, we assessed the sensitivity of mesenteric arteries from chow-fed mice to vasoconstriction and vasodilation (Fig. 5). Exposure to a high potassium PSS produced a contraction, which was not affected by the presence of mPVAT, in mesenteric arteries isolated from either male or female mice (Supplementary Fig. 1A-B). As anticipated, the presence of mPVAT reduced the sensitivity (EC_50_) of mesenteric arteries from both male and female mice to phenylephrine (PE), but did not alter the maximal contractile response (% Emax) (Fig. 5A, B; Supplementary Table 7). In both sexes, the presence of mPVAT had no effect on the relaxation of mesenteric arteries induced by either acetylcholine (ACh) (Fig. 5C, D) or the direct nitric oxide donor, sodium nitroprusside (SNP) (Fig. 5E, F; Supplementary Table 7).

### In obesity, the anti-contractile effect of mPVAT is lost in mesenteric arteries from female but not male mice

**Fig. 6:**
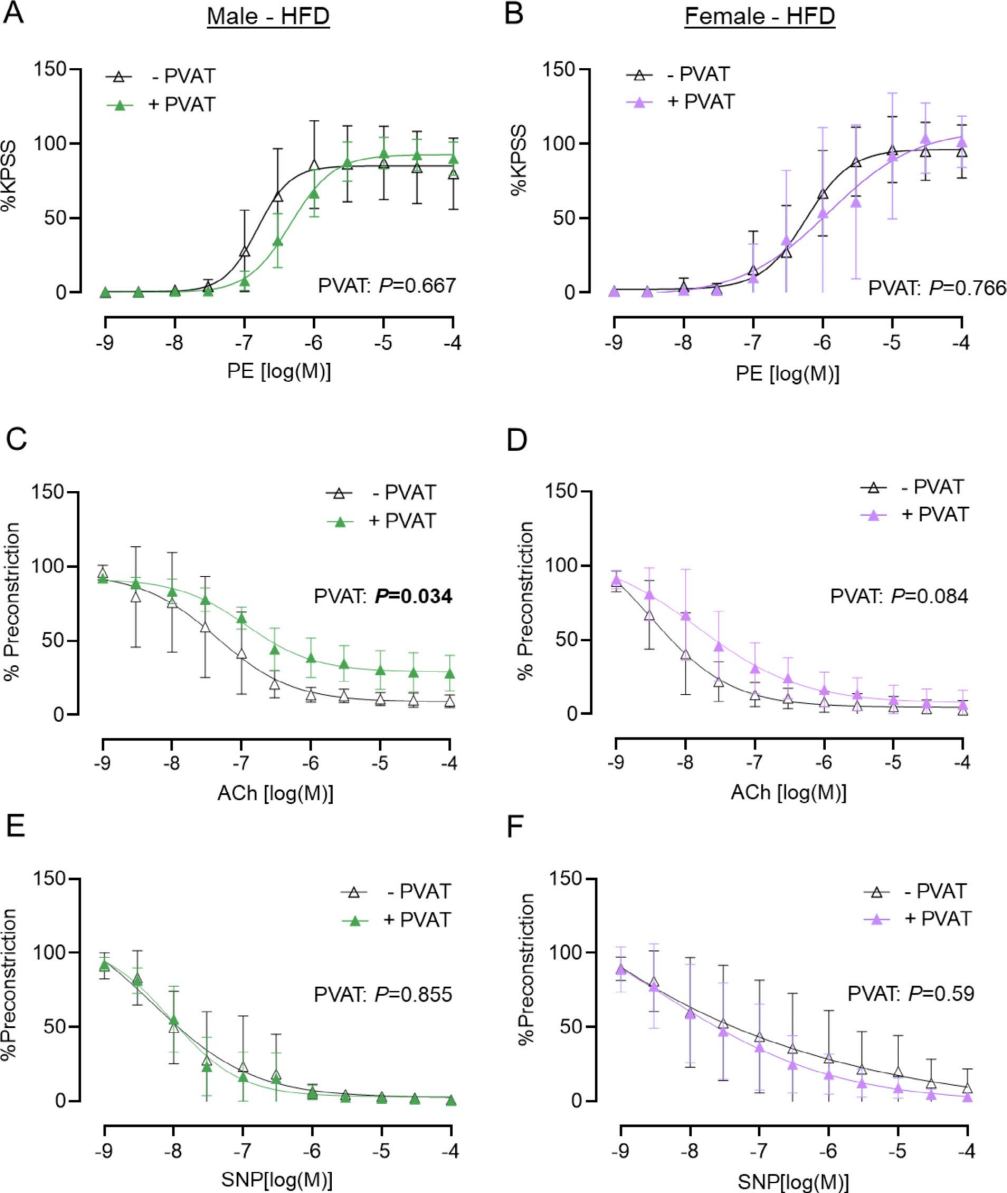
In mesenteric arteries from HFD-fed mice, obese mPVAT results in loss of anti-contractile effect in female mice, and impaired response to relaxation in male mice. Second order mesenteric arteries were dissected from male (left-hand panels **A**, **C**, **E**; green) and female (right-hand panels **B**, **D**, **F**; purple) C57Bl/6J mice after 16 weeks of HFD diet. PVAT was either removed (-PVAT; white symbol) or left intact (+ PVAT; colour symbol) and vessels were mounted onto a wire myograph. Concentration-response curves to phenylephrine (PE) (**A**, **B**), acetylcholine (ACh) (**C**, **D**) and sodium nitroprusside (SNP) (**E**, **F**) were obtained. For PE, contraction was normalised to the maximum response induced by a high potassium physiological salt solution (KPSS). For ACh and SNP, relaxation was normalised to a percentage of each vessel’s response to a half maximal concentration of PE (%Preconstriction). All concentration-response curves were generated by non-linear regression. LogEC 50 /IC 50 and %Emax/%Preconstriction were calculated for each concentration-response curve (Supplementary Table 7). Data are group mean ± SD (N = 6–8 per group), analysed by repeated measures two-way ANOVA. P < 0.05 was considered significant.

In mesenteric arteries from obese mice, the contractile response to depolarisation with extracellular potassium was not affected by the absence of mPVAT (Supplementary Fig. 1C-D). In obese male mice mPVAT maintained an anti-contractile effect in mesenteric arteries, with PVAT-intact vessels displaying reduced sensitivity (EC_50_) to PE compared with vessels with PVAT removed (Fig. 6A; Supplementary Table 7). In vessels from obese female mice, the anti-contractile effect was lost completely, with no significant differences in the response to PE between mesenteric arteries with mPVAT intact or removed (Fig. 6B; Supplementary Table 7).

### In obesity, mPVAT impairs endothelium-mediated relaxation in male mice but not female mice

In obese male mice, mPVAT resulted in significant differences in the response of mesenteric arteries to ACh, with reduced sensitivity (IC_50_) and maximal relaxation (% Preconstriction) compared to vessels with mPVAT removed (Fig. 6C; Supplementary Table 7. In contrast, PVAT had no significant effect on the response of mesenteric arteries from obese female mice to ACh (Fig. 6D; Supplementary Table 7). Importantly, in both sexes, there was no change in the relaxation of vessels with or without PVAT in response to SNP (Fig. 6E, F; Supplementary Table 7), indicating that the effect of PVAT in obese male mice is mediated by the endothelium and not vascular smooth muscle.

### Ovariectomy does not exacerbate mPVAT response to HFD

**Fig. 7:**
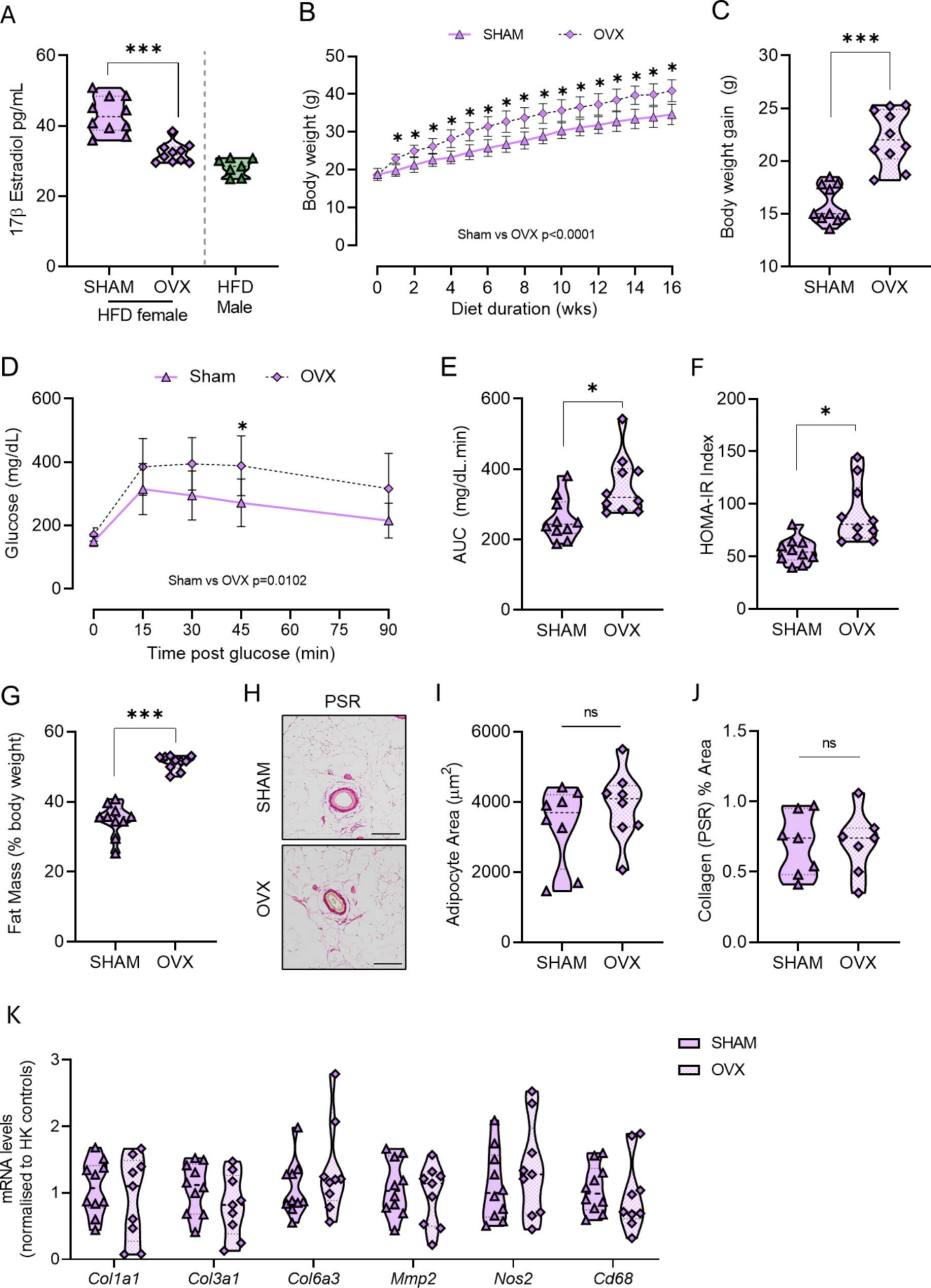
*Ovariectomy does not exacerbate the mesenteric PVAT (mPVAT) response to high fat diet (HFD)* Adult female mice underwent ovariectomy (OVX) surgery (purple triangles) or SHAM surgery (purple diamond), followed by 16 weeks of HFD. Serum 17β-estradiol levels, with inclusion of male mice administered HFD for 16 weeks (green triangles) as a comparison (**A**). Body weights were measured weekly (**B**). Weight gain (g) at 16 weeks was calculated from start of diet (**C**). Blood glucose levels during an IP glucose tolerance test performed at 15 weeks of diet; glucose was measured in plasma samples during a glucose tolerance test (**D**) and corresponding area under the curve (AUC) (**E**). HOMA-IR measure of insulin sensitivity was determined at 15 weeks of diet (**F**). Whole body fat mass as determined by TD-NMR (**G**). Data are mean ± SD (*n* = 10 per group). **B**,**D** analysed by RM 2-way ANOVA. **A**,**C**, **E**,**F**, **G** analysed by two-tailed unpaired t-test. **P < 0.01, **P < 0.001. Representative images of Picrosirius Red (PSR) staining in SHAM and OVX mice (**H**). Scale bar = 90 μm. Quantification of mean adipocyte area (**I**), and percent (%) PSR (collagen) staining (**J**). Data are expressed as mean ± SD (*n* = 8 per group), analysed by unpaired t-test. Transcript mRNA levels of *Col1a1*,* Col3a1*,* Col6a3*,* Mmp2*,* Nos2*,* Cd68* were determined by real-time PCR (**K**). Data are expressed as mean ± SD (*n* = 9–10). **I**,**J**, **K** analysed by unpaired two-tailed t-test

To determine whether the relative protection of female mice from HFD-induced mPVAT dysregulation and ECM remodelling was due to the presence of ovarian hormones, we performed ovariectomy (OVX) on female mice and compared their mPVAT phenotype under HFD to that in sham-operated HFD-fed female mice. OVX reduced serum levels of the most abundant estrogen 17β-estradiol compared with ovary-intact females (Fig. 7A). Notably, circulating 17β-estradiol levels were similar to those observed in male mice (Fig. 7A). Over the course of HFD administration, OVX mice gained more body weight than sham controls (Fig. 7B, C). Moreover, OVX resulted in exacerbated responses to glucose during a glucose tolerance test (Fig. 7D-E) and in measures of insulin resistance (Fig. 7F) and adiposity (Fig. 7G). Notably, histological assessment of mPVAT, which revealed that mPVAT from OVX females under HFD condition did not display significant adipocyte hypertrophy (Fig. 7H, I) or increased collagen deposition (Fig. 7J). This was further supported by assessment of ECM remodelling gene expression by qPCR, which demonstrated that OVX had no effect on the transcriptional response of those genes identified as being differentially expressed in mPVAT between obese male and obese female mice (Fig. 7K).

## Discussion

This work provides the first direct comparison of mPVAT between sexes in a murine model of diet-induced obesity, revealing crucial insights into the mechanisms of sex-specific adipose and vascular dysregulation. We demonstrate that obese male mice exhibit distinct ECM remodelling and increased collagen deposition in mPVAT, which is not observed in females, highlighting a novel sexually dimorphic pathway that may drive vascular pathologies in obesity. Importantly, our findings show that the relative protection observed in female mice from mPVAT ECM remodelling is independent of ovarian hormones, suggesting that other sex-specific factors contribute to this resilience. Moreover, compared to tPVAT, mPVAT displays greater transcriptional sensitivity to both diet and sex, underscoring its potential as a unqiue early indicator of pathological changes in obesity and a promising target for therapeutic intervention. These insights emphasise the need for personalised approaches in managing obesity-related vascular complications, particularly in addressing the unique cardiovascular risks in men and women.

To-date, most pre-clinical studies interrogating the link between obesity and PVAT have utilised male animals, meaning sex differences are relatively unexplored. Those that have investigated sex-specific response of PVAT have primarily used rats, with limited studies on mouse models of pathophysiology. Contreras and colleagues first demonstrated sexual dimorphism in healthy PVAT from rats, highlighting that female rats have significantly fewer adipose progenitor cells in both mPVAT and tPVAT compared to males [22], and subsequently that female rats have a greater number of CD4 and CD8 T-cells in their PVAT than male rats [23]. In obese rats, investigation of immune cell populations in mPVAT revealed further sex-specific differences in both T-cell and macrophage populations [24]. In mice, there is a paucity of data on the impact of sex on PVAT and vascular dysfunction in obesity. Gupte and colleagues demonstrated that 16-week administration of a 60% kcal fat HFD was sufficient to induce an increase (approx. 10 mmHg) in systolic blood pressure in male, but not female mice [25]. This was attributed to sex-specific differences in the expression of angiotensin-converting enzyme in white adipose tissue, but notably PVAT itself was not investigated. Here, we provide the first parallel comparisons of PVAT and vascular dysfunction in the mesentery, contrasting chow-fed vs. obese male and female mice.

Our RNA-seq data from PVAT highlight both the unique sensitivity of mPVAT to HFD-induced transcriptional changes, and also demonstrate the distinct sexually dimorphic responses of mPVAT in obesity. The marked increase in ECM remodelling pathways in mPVAT of obese male mice compared with obese female mice is supported by our RT-qPCR and histological data, and suggests significant changes to the PVAT microenvironment in obesity. Abnormal expression of ECM components in classical white adipose tissue is a hallmark of obesogenic tissue remodelling. In genetically obese *ob/ob* and diabetic *db/db* mice, transcript expression of multiple collagens (including I, III, and VI) are increased in visceral white adipose tissue compared to littermate controls [26]. Of particular interest is the change in expression of *Col6a3*. As one of the most abundant collagens in adipose tissue, *Col6* plays an essential role in shaping dysfunctional ECM [26, 27]. Moreover, in humans, *COL6A3* strongly correlates with the degree of hypoxia in white adipose tissue [28]. Excessive accumulation of *Col6a3* in mPVAT could disrupt normal ECM structure, increasing stiffness of the ECM scaffold, resulting in mechanical stress in the rapidly expanding adipose tissue [29]. Indeed, in mice, the absence of *Col6* leads to reduced rigidity, facilitating healthy expansion of white adipose tissue in both diet-induced obese and *ob/ob* mice [30]. Our finding that *Col6a3* was significantly lower in the mPVAT from obese female vs. obese male mice suggests that female mPVAT may be relatively protected from obesity-induced stiffness. Excessive collagen deposition in white adipose tissue has been observed in various animal models of metabolic diseases, and is consistent with our data showing increased collagen deposition in mPVAT from obese male, but not female, mice. To our knowledge, this is the first time collagen deposition has been assessed in mPVAT in response to obesity in male and female mice. This potential enhanced extracellular stiffness relative to the “normal” levels observed in chow-fed control mice may contribute to the impaired vasodilation mediated by mPVAT in obese male mice. A limitation of our work is that while we determined altered collagen deposition in response to high-fat diet, we did not assess if there were differences in collagen composition or cross-linking, aspects that would further enhance our understanding of the sexual dimorphic responses. Importantly, recognizing that male mPVAT is more susceptible to ECM remodelling in obesity could guide future pharmacological interventions targeting this pathway.

Despite the growing body of literature highlighting the functional role of PVAT on vascular tone, it is still routinely removed in most ex vivo vascular function studies. Identified as possessing anti-contractile effects under normal circumstances, various PVAT-produced factors including nitric oxide, hydrogen sulphide, angiotensin 1–7, adiponectin and insulin have all been implicated in the vasorelaxant effect it exerts on vessels [31, 32]. A number of research groups have reported, in both humans and rodents, a loss of PVAT’s anti-contractile effect in male obesity, including in aortic (thoracic and abdominal) [12, 13, 19] and mesenteric PVAT [33]. However, there are conflicting reports about the magnitude and sex-specific nature of this ‘anti-contractile effect loss’ [34]. Here, we show that male but not female mice appear relatively protected from obesity-induced loss of the anti-contractile effect of PVAT on mesenteric arteries. Supporting our findings on vasoconstriction, Victorio et al. also found that, when fed an alternative high-fat and high-sugar (45% kcal fat) diet for 12 weeks or 20 weeks, the anti-contractile effect of mPVAT was preserved in male mice. Conversely, in females on the same diet, the anticontractile effect of mPVAT was completely diminished [34]. In response to vasodilation, we found mPVAT impaired the ACh-induced relaxation in male but not female mice. While these results may appear paradoxical, they provide important insights into the underlying sex-specific vascular dysfunction and its implications for hypertension risk. Endothelial cell dysfunction, as indicated by the impaired response to ACh vasodilation in obese male mice, is an established early marker of vascular disease, which can contribute to arterial stiffness and increased vascular resistance. Therefore, endothelial cell dysfunction is often considered a better indicator of long-term vascular pathology, and suggests that female mice are relatively protected in this model. Our finding of mPVAT-mediated endothelial cell dysfunction in obese male mice, while obese female mice appear protected, is distinct from that observed by Victorio and colleagues who found female mice were more prone to obesity-induced endothelial cell dysfunction [34]. However, others have shown that the presence of PVAT reduces the maximal response of vessels to ACh-induced relaxation in obese male mice [13, 35]. Such differences between studies likely reflect differences in diet composition outlined, or in duration of HFD-feeding which ranges from 12 weeks to 8 months.

A notable finding was the depot-specific responses of PVAT between sexes. Multiple studies have highlighted that PVAT morphology depends on the anatomical location of the vessel. tPVAT has a similar phenotype to brown adipose tissue, while mPVAT shares a phenotype similar to white adipose tissue [36]. Our study to investigate both of these depots in response to obesity: carrying this out in both sexes. Our data demonstrate that under HFD conditions, mPVAT displayed markedly more DEGs between sexes than tPVAT (1271 vs. 56), and pathway analyses indicated significant enrichment for altered ECM remodelling pathways in mPVAT vs. altered regulation of insulin-like growth factor transport in tPVAT. Moreover, our data show that the magnitude of response to HFD is much greater in mPVAT vs. tPVAT, an effect that was evident in both sexes. This transcriptional heterogeneity is supported by another study that utilised RNA-seq to investigate regional responses of distinct PVAT depots to angiotensin II [18]. Together, this enhanced sensitivity suggests a more dynamic role for mPVAT in mediating responses to obesity, and suggests that future research should prioritise mPVAT when investigating obesity’s impact on vascular health.

To our knowledge, our study is also the first to investigate the effect of ovariectomy on the profile of mPVAT in response to obesity. We hypothesised that sex-hormone regulation of mPVAT may underlie its sexually dimorphic response to HFD. Estrogens can exert beneficial effects against the development of obesity, providing women with a healthier metabolic profile and conferring cardiovascular protection [37]. However, the effect of menopause and decreasing estrogen levels on mPVAT integrity is unknown. Despite reducing circulating levels of the primary estrogen 17β-estradiol, we did not find any changes in the transcriptional profile of mPVAT between OVX and sham-operated female mice under HFD conditions. However, given reports of estrogen production in adipose tissue [38], we cannot rule out that local levels within mPVAT were also reduced. Previous work in healthy rats has shown that OVX induces hypertrophy and inflammation in PVAT [39]. While we did not assess PVAT under normal chow-fed conditions in this model, we show that under HFD conditions OVX does not increase adipocyte size or collagen deposition in mPVAT. Interestingly, removal of ovarian hormones did result in increased weight gain over the course of HFD-feeding, and an increase in whole body adiposity, accompanied by worsened glucose tolerance and insulin resistance, indicating that these hormones likely play a more important role in classical white adipose tissue expansion and glucose homeostasis, rather than in ECM remodelling in PVAT. Notably, while it is clear that PVAT mass is increased in animal models and humans with obesity [40], the impact of sex on this increase is less clear. Future studies should determine if there is preferential expansion of distinct PVAT depots in response to obesity, and if this sexually dimorphic in nature. Importantly, this insight refines our understanding of hormonal influences on vascular health, moving beyond a simplistic focus on estrogen and opening avenues for exploring alternative mechanisms. One such alternative explanation for the lack of estrogen ‘protection’ in mPVAT is that the adverse phenotype is driven by androgens. In women with polycystic ovary syndrome, high levels of testosterone in adipose tissue are associated with disruptions to insulin action and adipokine secretion, markers of adipose dysfunction [41, 42, 43]. While there are reports indicating testosterone improves adipose function, although this is in obese hypogondal men [44, 45]. Crucially, studies assessing obesity-associated PVAT and vascular function following androgen removal (orchidectomy) in males and/or androgen administration in healthy females are lacking, and future investigations should prioritise the androgen/estrogen balance, both in the circulation but also at a tissue level, rather than focusing on sex steroids in isolation.

An important caveat in our study is that we did not observe any changes in blood pressure in our mice under HFD conditions, despite similar duration and dietary fat composition to others [25] (58% kcal fat vs. 60% kcal fat). This may be due to our method of blood pressure measurement, using the less sensitive tail-cuff plethysmography compared to the gold-standard radiotelemetry approaches used by Gupte and colleagues. However, it may also be due to crucial subtle differences in dietary composition and/or mice sub strain. Gupte and colleagues used an alternative obesogenic diet from Research Diets (D12492 vs. D12331 used here) and while fat content is similar (60% kcal fat vs. 58% kcal fat), sodium content is higher in D12492 (0.4% vs. 0.25%), which may have influenced the hypertensive response observed in that study [25]. Similarly, the previous study used a distinct strain of mice (C57Bl/6NTac vs. C57Bl/6JOlaHsd used here), which is known to be more sensitive to dietary salt [46] and may therefore have been more likely to respond to a HFD with a higher sodium content. A more recent study by Victorio and colleagues compared two different obesogenic diets in C57Bl/6J mice, and found that while both 45% kcal fat and 60% kcal fat increased adiposity and fasting glucose at 3 and 5 months dietary duration, there was no increase in blood pressure (as measured by tail-cuff plethysmography) [34]. Nonetheless, in our study we did observe an elevated heart rate in obese mice of both sexes, which may reflect a compensatory mechanism to maintain cardiac output, or a subclinical vascular changes such as those we observed in our myography analyses. A further limitation of our work is that we did not assess the impact of OVX on vascular responses in mesenteric arteries. In healthy rats, mesenteric resistance arteries from ovariectomized compared with sham-operated rats had dysfunctional responses to ACh, including decreased ACh-induced endothelium-dependent relaxation [47]. The presence of PVAT was able to attenuate this impairment in sham-operated control rats, but not in OVX rats, suggesting that ovarian hormones have a beneficial role in PVAT-mediated vascular function [47]. It may be that, despite not observing any transcriptional or morphological changes in mPVAT from our OVX mice, assessment of vascular responses may have revealed differences. Future studies should seek to confirm these findings in obese models and also in mice.

In conclusion, these studies present substantial evidence of multifaceted sex differences in the response of PVAT to diet-induced obesity. Paired with our vascular function studies, our transcriptomic analysis highlight key differences in the ECM remodelling of mPVAT between male and female mice under obese conditions and suggest a potential mechanistic link between sex-specific PVAT dysfunction and vascular dysfunction. This has important implications for investigating sex-specific treatments and pharmacological targets for obesity-induced vascular complications.

## Electronic supplementary material

Supplementary Material 1

## Data Availability

Data are available upon request or from the University of Edinburgh DataShare Repository: https://datashare.ed.ac.uk.
