# Supplementary material for: Obesity-induced mesenteric PVAT remodelling is sexually dimorphic, but not driven by ovarian hormones: Short title: Obesity induces sex-specific responses in mesenteric PVAT
Source: Cardiovasc Diabetol. 2025 Jan 24;24:39. doi: 10.1186/s12933-025-02596-w (PMC11762466; doi:10.1186/s12933-025-02596-w)
Supplement: Supplementary file 1 — Supplementary Material 1 [file 12933_2025_2596_MOESM1_ESM.docx]

# Supplementary Material

|  |  |  |
| --- | --- | --- |
| **Gene Name** | **Forward Primer 5' - 3'** | **Reverse Primer 5' - 3'** |
| *18s (Rna18s)* | AAACGGCTACCACATCCAAG | CCTCCAATGGATCCTCGTTA |
| *Cd68* | GGGGCTCTTGGGAACTACAC | GTACCGTCACAACCTCCCTG |
| *Col1a1* | ATCAGCCCAAACCCCAAGGA | GCAGGAAGGTCAGCTGGA |
| *Col3a1* | TGGTGCTAAGGGTGAGGTTG | AATGCCAGCTGGACCCATTT |
| *Col6a3* | TTGGAGCTTCCAGTAAAATATGC | TCTCCTTTTCTGAGGTTGAGAAG |
| *Col12a1* | TGTTTGCGCACGTGCTTATT | CTCCTGTATGATGCCGACCC |
| *Hprt* | TCCTCCTCAGACCGCTTTT | CCTGGTTCATCATCGCTAATC |
| *Mmp2* | TCTGGGGACAAGTTCTGGAG | AGAAATAGCTGTGACCACCAC |
| *Nos2* | CACCTTGGAGTTCACCCAGT | ACCACTCGTACTTGGGATGC |
| *Tbp* | GGGAGAATCATGGACCAGAA | GATGGGAATTCCAGGAGTCA |

## Supplementary Table 1. Murine forward and reverse primer sequences for RT-qPCR.

| **Comparison** | **No. DEGs (Upregulated/downregulated)** |
| --- | --- |
| Chow female vs male | 0 |
| HFD vs chow female | 1493 (582/911) |
| HFD vs chow male | 3474 (1925/1549) |
| HFD female vs male | 1271 (483/788) |

## Supplementary Table 2. Summary of differentially expressed genes (DEGs) in mesenteric PVAT for each comparison at adjusted P<0.05 (adjusted for multiple testing by controlling for the false discovery rate).

| **Reactome Pathway** | **Differentially Expressed Genes** | **S** | **N** | **S/N (%)** | **Z-score** | **P (adj.)** |
| --- | --- | --- | --- | --- | --- | --- |
| NRAGE signals death through JNK | *Akap13, Arhgef10, Arhgef2, Arhgef3, Arhgef40, Arhgef6, Prex1, Rasgrf2, Vav3* | 9 | 49 | 18.37 | 1.286 | 1.29E-02 |
| Death Receptor Signalling | *Akap13, Arhgef10, Arhgef2, Arhgef3, Arhgef40, Arhgef6, Birc2, Madd, Nfkbia, Prex1, Rasgrf2, Tnfaip3, Ubb, Vav3* | 14 | 122 | 11.48 | 1.268 | 1.29E-02 |
| Cell death signalling via NRAGE, NRIF and NADE | *Akap13, Arhgef10, Arhgef2, Arhgef3, Arhgef40, Arhgef6, Prex1, Rasgrf2, Ubb, Vav3* | 10 | 67 | 14.93 | 1.222 | 1.29E-02 |
| p75 NTR receptor-mediated signalling | *Akap13, Arhgef10, Arhgef2, Arhgef3, Arhgef40, Arhgef6, Nfkbia, Prex1, Rasgrf2, Ubb, Vav3* | 11 | 82 | 13.41 | 1.215 | 1.29E-02 |
| Innate Immune System | *4930486L24Rik, Adgre5, Apaf1, App, Atp6v1c2, B2m, Birc2, C1qa, C1qb, C1ra, Cd47, Cfp, Cracr2a, Cybb, Dsn1, Ear2, Fcgr2b, Gsn, Hexb, Irf7, Jup, Klrk1, Lta4h, Lyn, Malt1, Manba, Nfam1, Nfkbia, Nod1, Nos1, Padi2, Panx1, Pld4, Pnp, Pros1, Ptprj, S100b, Serpinb6a, Siglece, Slc2a3, Slc44a2, Stat6, Tlr7, Tlr8, Tnfaip3, Ubb, Vav3, Wipf1* | 48 | 828 | 5.8 | 1.668 | 1.29E-02 |
| DDX58/IFIH1-mediated induction of interferon-alpha | *App, Irf7, Nfkbia, S100b, Tnfaip3, Ubb* | 6 | 29 | 20.69 | 1.114 | 4.40E-02 |
| Immune System | *4930486L24Rik, Adgre5, Apaf1, App, Atp6v1c2, B2m, Birc2, C1qa, C1qb, C1ra, Cd47, Cd74, Cfp, Col1a1, Cracr2a, Csf1r, Cybb, Dsn1, Dtx3l, Ear2, Fbxo21, Fcgr2b, Gsn, Hexb, Il1rn, Il2rb, Il34, Il4ra, Irf7, Jup, Klhl3, Klrk1, Lifr, Lta4h, Lyn, Malt1, Manba, Mrc1, Nfam1, Nfkbia, Nod1, Nos1, Padi2, Panx1, Pdcd1lg2, Pias1, Pld4, Pnp, Pros1, Ptk2b, Ptprj, Rap1gap2, Rnasel, Rnf19b, S100b, Sec24d, Serpinb6a, Siglece, Slc2a3, Slc44a2, Spsb1, Stat6, Tlr7, Tlr8, Tnfaip3, Tnfrsf14, Ubb, Vav3, Vhl, Wipf1* | 70 | 1440 | 4.86 | 1.845 | 4.70E-02 |
| Classical antibody-mediated complement activation | *C1qa, C1qb, C1ra* | 3 | 6 | 50 | 1.225 | 6.98E-02 |
| O-glycosylation of TSR domain-containing proteins | *Adamts10, Adamts15, Adamtsl2, Adamtsl4, Cfp, Thbs2* | 6 | 34 | 17.65 | 1.029 | 7.22E-02 |
| Rho GTPase cycle | *Akap13, Arhgef10, Arhgef2, Arhgef3, Arhgef40, Arhgef6, Prex1, Ralbp1, Rasgrf2, Rhobtb1, Srgap2, Vav3* | 12 | 127 | 9.45 | 1.065 | 8.48E-02 |
|  |  |  |  |  |  |  |
| ChREBP activates metabolic gene expression | *Acaca, Acacb, Acly, Agpat1, Fasn, Mlx, Mlxipl* | 7 | 7 | 100 | -2.646 | 1.30E-07 |
| Fatty acid metabolism | *Acaca, Acacb, Acadvl, Acbd6, Acly, Acot11, Acox2, Acsf2, Acsf3, Acsl3, Crat, Decr1, Echs1, Ehhadh, Elovl3, Elovl5, Elovl6, Fasn, Ggt5, Gpx4, Hadh, Hadha, Hadhb, Hsd17b12, Mid1ip1, Ndufab1, Nudt7, Pcca, Prkaa2, Ptges, Ptges2, Slc25a1, Slc25a20, Tecr, Them4, Thrsp* | 36 | 161 | 22.36 | -2.837 | 2.70E-11 |
| Pyruvate metabolism and TCA cycle | *Aco2, Bsg, Cs, Dlat, Dld, Fahd1, Idh3b, Idh3g, Ldha, Me1, Pdha1, Pdhb, Pdhx, Pdk1, Sdha, Sdhb, Sdhd, Slc16a1, Sucla2, Vdac1* | 20 | 47 | 42.55 | -2.917 | 1.32E-11 |
| Respiratory electron transport | *Coq10a, Ndufa10, Ndufa12, Ndufa5, Ndufa6, Ndufa8, Ndufa9, Ndufab1, Ndufaf3, Ndufaf4, Ndufb5, Ndufb6, Ndufb8, Ndufb9, Ndufs1, Ndufs2, Ndufs3, Ndufs4, Ndufs6, Ndufv1, Ndufv2, Ndufv3* | 22 | 52 | 42.31 | -3.051 | 1.27E-12 |
| Cholesterol biosynthesis | *Acat2, Cyp51, Dhcr7, Fdft1, Fdps, Hsd17b7, Idi1, Lss, Msmo1, Mvd, Nsdhl, Pmvk, Sc5d, Sqle, Tm7sf2* | 15 | 24 | 62.5 | -3.062 | 1.17E-11 |
| Complex I biogenesis | *Ndufa10, Ndufa12, Ndufa5, Ndufa6, Ndufa8, Ndufa9, Ndufab1, Ndufaf3, Ndufaf4, Ndufb5, Ndufb6, Ndufb8, Ndufb9, Ndufs1, Ndufs2, Ndufs3, Ndufs4, Ndufs6, Ndufv1, Ndufv2, Ndufv3* | 21 | 47 | 44.68 | -3.063 | 1.27E-12 |
| Metabolism of lipids | *Aacs, Acaca, Acacb, Acadvl, Acat2, Acbd6, Acer1, Acly, Acot11, Acox2, Acp6, Acsf2, Acsf3, Acsl3, Agpat1, Agpat2, Agpat3, Chpt1, Cidea, Crat, Cyp51, Ddhd2, Decr1, Dgat1, Dhcr7, Echs1, Ehhadh, Elovl3, Elovl5, Elovl6, Fabp5, Fasn, Fdft1, Fdps, Fdx1, Gde1, Ggt5, Gpd1, Gpd2, Gpx4, Hadh, Hadha, Hadhb, Hsd11b1, Hsd17b12, Hsd17b7, Hsd3b7, Idi1, Lss, Mid1ip1, Mogat1, Mogat2, Msmo1, Mvd, Ndufab1, Nsdhl, Nudt7, Ormdl3, Pcca, Pcyt2, Phospho1, Pi4k2a, Pik3cb, Pmvk, Pnpla3, Prkaa2, Ptges, Ptges2, Sc5d, Sgms2, Slc25a1, Slc25a20, Sqle, Srd5a1, Srebf1, Stard10, Tecr, Them4, Thrsp, Tm7sf2* | 80 | 562 | 14.23 | -3.375 | 2.16E-13 |
| Respiratory electron transport, ATP synthesis | *Atp5a1, Atp5b, Atp5c1, Atp5f1, Atp5g2, Atp5g3, Atp5j, Atp5j2, Atp5k, Coq10a, Ndufa10, Ndufa12, Ndufa5, Ndufa6, Ndufa8, Ndufa9, Ndufab1, Ndufaf3, Ndufaf4, Ndufb5, Ndufb6, Ndufb8, Ndufb9, Ndufs1, Ndufs2, Ndufs3, Ndufs4, Ndufs6, Ndufv1, Ndufv2, Ndufv3, Ucp3* | 32 | 72 | 44.44 | -3.771 | 2.38E-19 |
| TCA cycle and respiratory electron transport | *Aco2, Atp5a1, Atp5b, Atp5c1, Atp5f1, Atp5g2, Atp5g3, Atp5j, Atp5j2, Atp5k, Bsg, Coq10a, Cs, Dlat, Dld, Fahd1, Idh3b, Idh3g, Ldha, Me1, Ndufa10, Ndufa12, Ndufa5, Ndufa6, Ndufa8, Ndufa9, Ndufab1, Ndufaf3, Ndufaf4, Ndufb5, Ndufb6, Ndufb8, Ndufb9, Ndufs1, Ndufs2, Ndufs3, Ndufs4, Ndufs6, Ndufv1, Ndufv2, Ndufv3, Pdha1, Pdhb, Pdhx, Pdk1, Sdha, Sdhb, Sdhd, Slc16a1, Sucla2, Ucp3, Vdac1* | 52 | 119 | 43.7 | -4.767 | 8.23E-32 |
| Metabolism | *Aacs, Acaca, Acacb, Acadvl, Acat2, Acbd6, Acer1, Acly, Aco2, Acot11, Acox2, Acp6, Acsf2, Acsf3, Acsl3, Acss2, Adh1, Adi1, Agl, Agpat1, Agpat2, Agpat3, Ak2, Akr7a5, Alad, Aldh2, Aldh4a1, Aldoa, Apoc3, Aspg, Atp5a1, Atp5b, Atp5c1, Atp5f1, Atp5g2, Atp5g3, Atp5j, Atp5j2, Atp5k, B3gat3, Bsg, Car5b, Chpt1, Cidea, Cmpk1, Coasy, Comt, Coq10a, Coq3, Crat, Cs, Cspg4, Cspg5, Cyb5b, Cyb5r3, Cyp51, Ddhd2, Ddo, Decr1, Dgat1, Dhcr7, Dlat, Dld, Dpyd, Echs1, Ehhadh, Elovl3, Elovl5, Elovl6, Eno1, Eno1b, Entpd5, Fabp5, Fahd1, Fasn, Fdft1, Fdps, Fdx1, Gale, Gapdh, Gbe1, Gcat, Gclm, Gde1, Ggt5, Glrx5, Gng12, Gpd1, Gpd2, Gpi1, Gpt, Gpx4, Gsr, Gsto1, Gyg, Gys1, Hadh, Hadha, Hadhb, Hsd11b1, Hsd17b12, Hsd17b7, Hsd3b7, Idh3b, Idh3g, Idi1, Impa2, Isca1, Isyna1, Kcnb1, Ldha, Lss, Marc1, Marc2, Mdh1, Me1, Mgst2, Mid1ip1, Mlx, Mlxipl, Mogat1, Mogat2, Msmo1, Mvd, Nat8l, Ndufa10, Ndufa12, Ndufa5, Ndufa6, Ndufa8, Ndufa9, Ndufab1, Ndufaf3, Ndufaf4, Ndufb5, Ndufb6, Ndufb8, Ndufb9, Ndufs1, Ndufs2, Ndufs3, Ndufs4, Ndufs6, Ndufv1, Ndufv2, Ndufv3, Nmnat3, Nos3, Nsdhl, Nudt4, Nudt5, Nudt7, Ormdl3, Paics, Pank1, Parp6, Pcca, Pcyt2, Pdha1, Pdhb, Pdhx, Pdk1, Pdzd11, Pfkl, Pfkm, Pgam1, Pgd, Pgk1, Phospho1, Pi4k2a, Pik3cb, Pipox, Pkm, Pmvk, Pnpla3, Ppa1, Ppcs, Ppip5k1, Prkaa2, Prkaca, Psph, Ptges, Ptges2, Pycrl, Pygl, Qdpr, Rdh11, Rpia, Sardh, Sc5d, Sdha, Sdhb, Sdhd, Sgms2, Shpk, Slc16a1, Slc19a1, Slc25a1, Slc25a20, Slc25a32, Slc37a4, Slc46a1, Sord, Sqle, Srd5a1, Srebf1, Stard10, Sucla2, Taldo1, Tecr, Them4, Thrsp, Tkt, Tm7sf2, Txn2, Uck1, Uck2, Ucp3, Ugp2, Uros, Vdac1* | 221 | 1558 | 14.18 | -5.599 | 1.03E-46 |

## Supplementary Table 3: Reactome Pathways significantly enriched in mesenteric PVAT HFD female vs chow female. Individual Reactome pathways were assessed for significant enrichment by assessing whether the number (S) of significantly differentially expressed genes within a pathway is more than expected by chance given the total number (N) of genes. Adjusted p-value (P (adj.)) was determined using a hypergeometric test and corrected for tests over multiple pathways using the Benjamini-Hochberg procedure. Z-score is calculated as $Z=(Su-Sd)/\surd N$, where Su and Sd are the number of significant upregulated and downregulated genes in the pathway respectively.

| **Reactome Pathway** | **Differentially Expressed Genes** | **S** | **N** | **S/N (%)** | **Z-score** | **P (adj.)** |
| --- | --- | --- | --- | --- | --- | --- |
| Extracellular matrix organization | *Adam10, Adam12, Adam19, Adam8, Adamts2, Adamts4, App, Bgn, Bmp1, Capn1, Capn12, Cd44, Ceacam2, Col12a1, Col14a1, Col15a1, Col16a1, Col18a1, Col1a1, Col1a2, Col26a1, Col28a1, Col3a1, Col4a1, Col4a2, Col4a5, Col5a1, Col5a2, Col6a1, Col6a2, Col6a6, Col8a1, Colgalt1, Ctsk, Ctss, Ddr1, Dst, Eln, F11r, Fbln2, Fbln5, Fn1, Hspg2, Icam2, Itga6, Itgax, Itgb1, Itgb2, Jam2, Lama5, Lamb3, Lox, Loxl1, Loxl2, Loxl3, Ltbp3, Lum, Mfap3, Mfap4, Mmp11, Mmp12, Mmp13, Mmp14, Mmp2, Nid1, P3h1, P3h2, P3h3, Pcolce, Pcolce2, Pdgfb, Pecam1, Plec, Sdc1, Sdc3, Sparc, Spp1, Timp1, Tnc, Tnr, Vtn* | 81 | 248 | 32.66 | 5.144 | 1.14E-17 |
| Collagen formation | *Adamts2, Bmp1, Col12a1, Col14a1, Col15a1, Col16a1, Col18a1, Col1a1, Col1a2, Col26a1, Col28a1, Col3a1, Col4a1, Col4a2, Col4a5, Col5a1, Col5a2, Col6a1, Col6a2, Col6a6, Col8a1, Colgalt1, Ctss, Dst, Itga6, Lamb3, Lox, Loxl1, Loxl2, Loxl3, Mmp13, P3h1, P3h2, P3h3, Pcolce, Pcolce2, Plec* | 37 | 79 | 46.84 | 4.163 | 4.70E-13 |
| Signal Transduction | *Abhd12, Abl1, Abr, Acvr1b, Acvrl1, Adam10, Adam17, Adcy4, Adcy7, Adcyap1r1, Adgre1, Adm, Adora2a, Agtr1a, Akap13, Akt3, Aldh1a2, Aldh1a3, Anxa1, Aplnr, App, Arap3, Arhgap1, Arhgap10, Arhgap11a, Arhgap17, Arhgap18, Arhgap22, Arhgap23, Arhgap25, Arhgap27, Arhgap31, Arhgap33, Arhgap39, Arhgap6, Arhgef10, Arhgef10l, Arhgef15, Arhgef17, Arhgef2, Arhgef26, Arhgef40, Arhgef5, Arhgef6, Arhgef7, Arpc1b, Arrb1, Atp6v0d2, Atp6v1c2, Aurkb, Bcl2l11, Bcl9l, Bdnf, Boc, Bub1b, C3ar1, Casp2, Casp8, Cbx2, Ccr5, Cdc42ep2, Cdc42ep3, Cdh5, Cenpa, Cenpe, Chn2, Chrm4, Cilp, Cit, Clip1, Clip3, Col1a1, Col1a2, Col3a1, Col4a1, Col4a2, Col4a5, Col5a1, Col5a2, Col6a1, Col6a2, Col6a6, Cpt1a, Csf2ra, Csf2rb, Ctnnb1, Ctnnbip1, Ctnnd1, Cxcl2, Cxcl9, Cybb, Cyfip1, Daam1, Dab2ip, Daglb, Dgke, Dgkh, Dgki, Dgkz, Diaph1, Diaph3, Dll1, Dll4, Dok1, Dsn1, Dusp10, Dusp3, Dusp8, Dusp9, Ece1, Ect2, Elmo1, Epor, Eps15, Erbb2, Ercc6l, Evc, Evl, F11r, F2r, F2rl3, Fes, Ffar2, Flna, Flt1, Flt4, Fmnl1, Fmnl3, Fn1, Fos, Fstl1, Fstl3, Fyn, Fzd6, Gab1, Gabbr1, Gabbr2, Gipr, Gna12, Gnai2, Gnao1, Gnb4, Gng2, Gpc1, Gpc6, Gpnmb, Gpr4, Gpr68, Gpsm1, Grap, Grin2d, Grk5, Hif1a, Hspg2, Ikbkg, Il17rd, Il1rl1, Il2ra, Incenp, Inhbb, Iqgap1, Iqgap2, Irak4, Itgb1, Jag2, Jak3, Jun, Kalrn, Kdm1a, Kitl, Kremen1, Ksr2, Lama5, Lamb3, Lats2, Lpar3, Lyn, Mad1l1, Madd, Maml1, Maml2, Maml3, Mamld1, Map3k11, Mapk7, Matk, Mecom, Mmp2, Mrtfa, Myh9, Myo7a, Nckap1l, Ncor2, Nln, Notch3, Notch4, Npy, Nrp1, Nrp2, Numb, Olfr1396, Olfr558, P2ry1, P2ry2, P2ry6, Pde1b, Pde2a, Pde3a, Pde4b, Pde7a, Pde7b, Pdgfb, Pdgfrb, Pgf, Phlpp1, Pik3ap1, Pik3cg, Pik3r3, Pik3r5, Pik3r6, Pip4k2a, Pip5k1c, Pkn1, Pkn3, Plcg1, Plekhg2, Polr2a, Ppp2r5c, Prex1, Prex2, Prkag3, Prkcd, Prkcg, Prkch, Psap, Psen2, Ptafr, Ptk2b, Ptprk, Racgap1, Ralbp1, Ralgds, Ramp2, Ranbp10, Rapgef1, Rara, Rasa4, Rasgrf2, Rasgrp4, Rbl1, Rbpj, Reep4, Ret, Rgs1, Rgs12, Rgs4, Rgs5, Rhoc, Rps6ka2, Rtp3, S1pr1, S1pr2, Sdc1, Sdc3, Sel1l, Sgo2a, Sh2b3, Shc2, Smad1, Smad6, Sox13, Sox17, Sox7, Spc25, Spp1, Sppl2a, Spred2, Spred3, Spry1, Sptbn1, Src, Srgap2, Stard8, Stat6, Sufu, Syk, Tcirg1, Tec, Tek, Tgif1, Thbs4, Tjp1, Tjp2, Tle2, Tln1, Tnfaip3, Tns3, Tyk2, Vav3, Vegfc, Vegfd, Wipf1, Wwtr1* | 303 | 1914 | 15.83 | 6.926 | 2.48E-11 |
| Assembly of collagen fibrils | *Bmp1, Col12a1, Col14a1, Col15a1, Col18a1, Col1a1, Col1a2, Col3a1, Col4a1, Col4a2, Col4a5, Col5a1, Col5a2, Col6a1, Col6a2, Col6a6, Col8a1, Ctss, Dst, Itga6, Lamb3, Lox, Loxl1, Loxl2, Loxl3, Mmp13, Plec* | 27 | 54 | 50 | 3.674 | 2.66E-10 |
| Collagen biosynthesis and modifying enzymes | *Adamts2, Bmp1, Col12a1, Col14a1, Col15a1, Col16a1, Col18a1, Col1a1, Col1a2, Col26a1, Col28a1, Col3a1, Col4a1, Col4a2, Col4a5, Col5a1, Col5a2, Col6a1, Col6a2, Col6a6, Col8a1, Colgalt1, P3h1, P3h2, P3h3, Pcolce, Pcolce2* | 27 | 58 | 46.55 | 3.545 | 1.86E-09 |
| Integrin cell surface interactions | *Cd44, Col16a1, Col18a1, Col1a1, Col1a2, Col3a1, Col4a1, Col4a2, Col4a5, Col5a1, Col5a2, Col6a1, Col6a2, Col6a6, Col8a1, F11r, Fn1, Hspg2, Icam2, Itga6, Itgax, Itgb1, Itgb2, Jam2, Lum, Pecam1, Spp1, Tnc, Vtn* | 29 | 69 | 42.03 | 3.491 | 6.00E-09 |
| Degradation of the extracellular matrix | *Adam10, Adam8, Adamts4, Bmp1, Capn1, Capn12, Cd44, Col12a1, Col15a1, Col18a1, Col1a1, Col1a2, Col3a1, Col4a1, Col4a2, Col4a5, Col5a1, Col5a2, Col6a1, Col6a2, Col6a6, Col8a1, Ctsk, Ctss, Eln, Fn1, Hspg2, Lamb3, Mmp11, Mmp12, Mmp13, Mmp14, Mmp2, Nid1, Spp1, Timp1* | 36 | 113 | 31.86 | 3.387 | 2.47E-07 |
| Collagen chain trimerization | *Col12a1, Col14a1, Col15a1, Col16a1, Col18a1, Col1a1, Col1a2, Col26a1, Col28a1, Col3a1, Col4a1, Col4a2, Col4a5, Col5a1, Col5a2, Col6a1, Col6a2, Col6a6, Col8a1* | 19 | 38 | 50 | 3.082 | 3.24E-07 |
| Rho GTPase cycle | *Abr, Akap13, Arap3, Arhgap1, Arhgap10, Arhgap11a, Arhgap17, Arhgap18, Arhgap22, Arhgap23, Arhgap25, Arhgap27, Arhgap31, Arhgap33, Arhgap39, Arhgap6, Arhgef10, Arhgef10l, Arhgef15, Arhgef17, Arhgef2, Arhgef26, Arhgef40, Arhgef5, Arhgef6, Arhgef7, Chn2, Ect2, Kalrn, Plekhg2, Prex1, Racgap1, Ralbp1, Rasgrf2, Rhoc, Srgap2, Stard8, Vav3* | 38 | 127 | 29.92 | 3.372 | 4.94E-07 |
| Collagen degradation | *Col12a1, Col15a1, Col18a1, Col1a1, Col1a2, Col3a1, Col4a1, Col4a2, Col4a5, Col5a1, Col5a2, Col6a1, Col6a2, Col6a6, Col8a1, Ctsk, Mmp11, Mmp12, Mmp13, Mmp14, Mmp2* | 21 | 49 | 42.86 | 3 | 1.25E-06 |
| Translation | *Apeh, Aurkaip1, Eif3e, Eif3i, Eif3m, Eif4a2, Eral1, Gfm1, Gfm2, Mrpl1, Mrpl10, Mrpl14, Mrpl16, Mrpl17, Mrpl18, Mrpl2, Mrpl20, Mrpl21, Mrpl22, Mrpl23, Mrpl28, Mrpl3, Mrpl30, Mrpl32, Mrpl34, Mrpl36, Mrpl38, Mrpl39, Mrpl42, Mrpl44, Mrpl46, Mrpl47, Mrpl49, Mrpl50, Mrpl51, Mrpl52, Mrpl55, Mrpl9, Mrps14, Mrps15, Mrps16, Mrps17, Mrps18a, Mrps18b, Mrps18c, Mrps21, Mrps23, Mrps24, Mrps25, Mrps26, Mrps27, Mrps30, Mrps31, Mrps35, Mrps36, Mrps5, Mrps7, Mrps9, Oxa1l, Ppa1, Ppa2, Ptcd3, Rps19, Rpsa, Srp14, Uba52* | 66 | 217 | 30.41 | -4.48 | 8.66E-17 |
| Pyruvate metabolism and TCA cycle | *Aco2, Bsg, Cs, Dlat, Dld, Fahd1, Fh1, Glo1, Hagh, Idh3b, Idh3g, L2hgdh, Ldha, Ldhb, Mdh2, Me1, Pdha1, Pdhb, Pdhx, Pdk1, Pdk2, Pdp2, Sdha, Sdhb, Sdhc, Sdhd, Slc16a1, Sucla2, Suclg1, Suclg2, Vdac1* | 31 | 47 | 65.96 | -4.522 | 3.55E-19 |
| Mitochondrial translation termination | *Aurkaip1, Eral1, Gfm2, Mrpl1, Mrpl10, Mrpl14, Mrpl16, Mrpl17, Mrpl18, Mrpl2, Mrpl20, Mrpl21, Mrpl22, Mrpl23, Mrpl28, Mrpl3, Mrpl30, Mrpl32, Mrpl34, Mrpl36, Mrpl38, Mrpl39, Mrpl42, Mrpl44, Mrpl46, Mrpl47, Mrpl49, Mrpl50, Mrpl51, Mrpl52, Mrpl55, Mrpl9, Mrps14, Mrps15, Mrps16, Mrps17, Mrps18a, Mrps18b, Mrps18c, Mrps21, Mrps23, Mrps24, Mrps25, Mrps26, Mrps27, Mrps30, Mrps31, Mrps35, Mrps36, Mrps5, Mrps7, Mrps9, Oxa1l, Ptcd3* | 54 | 85 | 63.53 | -5.857 | 8.32E-33 |
| Mitochondrial translation elongation | *Aurkaip1, Eral1, Gfm1, Mrpl1, Mrpl10, Mrpl14, Mrpl16, Mrpl17, Mrpl18, Mrpl2, Mrpl20, Mrpl21, Mrpl22, Mrpl23, Mrpl28, Mrpl3, Mrpl30, Mrpl32, Mrpl34, Mrpl36, Mrpl38, Mrpl39, Mrpl42, Mrpl44, Mrpl46, Mrpl47, Mrpl49, Mrpl50, Mrpl51, Mrpl52, Mrpl55, Mrpl9, Mrps14, Mrps15, Mrps16, Mrps17, Mrps18a, Mrps18b, Mrps18c, Mrps21, Mrps23, Mrps24, Mrps25, Mrps26, Mrps27, Mrps30, Mrps31, Mrps35, Mrps36, Mrps5, Mrps7, Mrps9, Oxa1l, Ptcd3* | 54 | 83 | 65.06 | -5.927 | 1.49E-33 |
| Mitochondrial translation | *Aurkaip1, Eral1, Gfm1, Gfm2, Mrpl1, Mrpl10, Mrpl14, Mrpl16, Mrpl17, Mrpl18, Mrpl2, Mrpl20, Mrpl21, Mrpl22, Mrpl23, Mrpl28, Mrpl3, Mrpl30, Mrpl32, Mrpl34, Mrpl36, Mrpl38, Mrpl39, Mrpl42, Mrpl44, Mrpl46, Mrpl47, Mrpl49, Mrpl50, Mrpl51, Mrpl52, Mrpl55, Mrpl9, Mrps14, Mrps15, Mrps16, Mrps17, Mrps18a, Mrps18b, Mrps18c, Mrps21, Mrps23, Mrps24, Mrps25, Mrps26, Mrps27, Mrps30, Mrps31, Mrps35, Mrps36, Mrps5, Mrps7, Mrps9, Oxa1l, Ptcd3* | 55 | 86 | 63.95 | -5.931 | 1.49E-33 |
| Complex I biogenesis | *Ecsit, Ndufa1, Ndufa10, Ndufa12, Ndufa13, Ndufa2, Ndufa3, Ndufa5, Ndufa6, Ndufa7, Ndufa8, Ndufa9, Ndufab1, Ndufaf1, Ndufaf3, Ndufaf4, Ndufaf5, Ndufaf7, Ndufb10, Ndufb11, Ndufb2, Ndufb3, Ndufb4, Ndufb5, Ndufb6, Ndufb7, Ndufb8, Ndufb9, Ndufc1, Ndufc2, Ndufs1, Ndufs2, Ndufs3, Ndufs4, Ndufs5, Ndufs6, Ndufs7, Ndufv1, Ndufv2, Ndufv3, Nubpl, Timmdc1* | 42 | 47 | 89.36 | -6.126 | 5.88E-36 |
| Respiratory electron transport | *Coq10a, Ecsit, Etfb, Etfdh, Ndufa1, Ndufa10, Ndufa12, Ndufa13, Ndufa2, Ndufa3, Ndufa5, Ndufa6, Ndufa7, Ndufa8, Ndufa9, Ndufab1, Ndufaf1, Ndufaf3, Ndufaf4, Ndufaf5, Ndufaf7, Ndufb10, Ndufb11, Ndufb2, Ndufb3, Ndufb4, Ndufb5, Ndufb6, Ndufb7, Ndufb8, Ndufb9, Ndufc1, Ndufc2, Ndufs1, Ndufs2, Ndufs3, Ndufs4, Ndufs5, Ndufs6, Ndufs7, Ndufv1, Ndufv2, Ndufv3, Nubpl, Timmdc1* | 45 | 52 | 86.54 | -6.24 | 3.83E-37 |
| Respiratory electron transport, ATP synthesis | *Atp5a1, Atp5b, Atp5c1, Atp5d, Atp5e, Atp5f1, Atp5g1, Atp5g2, Atp5g3, Atp5h, Atp5j, Atp5j2, Atp5k, Atp5l, Coq10a, Ecsit, Etfb, Etfdh, Ndufa1, Ndufa10, Ndufa12, Ndufa13, Ndufa2, Ndufa3, Ndufa5, Ndufa6, Ndufa7, Ndufa8, Ndufa9, Ndufab1, Ndufaf1, Ndufaf3, Ndufaf4, Ndufaf5, Ndufaf7, Ndufb10, Ndufb11, Ndufb2, Ndufb3, Ndufb4, Ndufb5, Ndufb6, Ndufb7, Ndufb8, Ndufb9, Ndufc1, Ndufc2, Ndufs1, Ndufs2, Ndufs3, Ndufs4, Ndufs5, Ndufs6, Ndufs7, Ndufv1, Ndufv2, Ndufv3, Nubpl, Timmdc1, Ucp3* | 60 | 72 | 83.33 | -7.071 | 6.01E-48 |
| TCA cycle and respiratory electron transport | *Aco2, Atp5a1, Atp5b, Atp5c1, Atp5d, Atp5e, Atp5f1, Atp5g1, Atp5g2, Atp5g3, Atp5h, Atp5j, Atp5j2, Atp5k, Atp5l, Bsg, Coq10a, Cs, Dlat, Dld, Ecsit, Etfb, Etfdh, Fahd1, Fh1, Glo1, Hagh, Idh3b, Idh3g, L2hgdh, Ldha, Ldhb, Mdh2, Me1, Ndufa1, Ndufa10, Ndufa12, Ndufa13, Ndufa2, Ndufa3, Ndufa5, Ndufa6, Ndufa7, Ndufa8, Ndufa9, Ndufab1, Ndufaf1, Ndufaf3, Ndufaf4, Ndufaf5, Ndufaf7, Ndufb10, Ndufb11, Ndufb2, Ndufb3, Ndufb4, Ndufb5, Ndufb6, Ndufb7, Ndufb8, Ndufb9, Ndufc1, Ndufc2, Ndufs1, Ndufs2, Ndufs3, Ndufs4, Ndufs5, Ndufs6, Ndufs7, Ndufv1, Ndufv2, Ndufv3, Nubpl, Pdha1, Pdhb, Pdhx, Pdk1, Pdk2, Pdp2, Sdha, Sdhb, Sdhc, Sdhd, Slc16a1, Sucla2, Suclg1, Suclg2, Timmdc1, Ucp3, Vdac1* | 91 | 119 | 76.47 | -8.342 | 3.58E-67 |
| Metabolism | *Aacs, Aasdhppt, Acaca, Acacb, Acad10, Acad8, Acads, Acadsb, Acadvl, Acat1, Acat2, Acly, Aco2, Acot13, Acot2, Acot4, Acox2, Acp6, Acsf3, Acsm5, Acss2, Acy1, Adh1, Adh5, Adi1, Adipoq, Adsl, Agl, Agpat1, Agpat2, Agpat3, Ak2, Akr7a5, Alad, Aldh2, Aldh4a1, Aldh6a1, Aldh7a1, Aldoa, Apoc3, Asns, Aspa, Aspg, Atp5a1, Atp5b, Atp5c1, Atp5d, Atp5e, Atp5f1, Atp5g1, Atp5g2, Atp5g3, Atp5h, Atp5j, Atp5j2, Atp5k, Atp5l, B3gat3, Bcat2, Bckdhb, Bckdk, Bphl, Bpnt1, Bsg, Car13, Car5b, Carnmt1, Cbr1, Cbr4, Cdo1, Cept1, Cers2, Ces1d, Chac2, Chpt1, Cidea, Coasy, Comt, Coq10a, Coq2, Coq3, Cpt2, Crat, Crls1, Crym, Cs, Cspg5, Cyb5a, Cyp26b1, Cyp2e1, Cyp2f2, Cyp51, Dbt, Dct, Ddhd2, Ddo, Decr1, Dgat1, Dhodh, Dlat, Dld, Dlst, Dpyd, Echs1, Eci1, Ecsit, Elovl6, Eno1, Entpd5, Etfb, Etfdh, Fah, Fahd1, Fasn, Fdft1, Fdx1, Fh1, Flad1, Fmo1, Fxn, Gale, Gapdh, Gbe1, Gcat, Gcdh, Gde1, Glo1, Gmps, Gpam, Gpd2, Gpi1, Gpx4, Grhpr, Gsr, Gsta3, Gstk1, Gstm2, Gstm4, Gstm5, Gstm7, Gsto1, Gsto2, Gstp1, Gstt1, Gyg, Gys1, H2-Ke6, Hadh, Hadha, Hadhb, Hagh, Hgsnat, Hibadh, Hibch, Hk2, Hs3st6, Hsd11b1, Hsd17b12, Hsd3b7, Idh3b, Idh3g, Idi1, Isca1, Itpa, Ivd, Kcnb1, Kdsr, Khk, L2hgdh, Ldha, Ldhb, Maob, Marc1, Marc2, Mcat, Mccc1, Mccc2, Mcee, Mdh1, Mdh2, Me1, Mecr, Mgst1, Mgst2, Mgst3, Mid1ip1, Mlxipl, Mmaa, Mmab, Mmadhc, Mmut, Mogat1, Mpst, Msmo1, Nampt, Nat8l, Naxe, Ndufa1, Ndufa10, Ndufa12, Ndufa13, Ndufa2, Ndufa3, Ndufa5, Ndufa6, Ndufa7, Ndufa8, Ndufa9, Ndufab1, Ndufaf1, Ndufaf3, Ndufaf4, Ndufaf5, Ndufaf7, Ndufb10, Ndufb11, Ndufb2, Ndufb3, Ndufb4, Ndufb5, Ndufb6, Ndufb7, Ndufb8, Ndufb9, Ndufc1, Ndufc2, Ndufs1, Ndufs2, Ndufs3, Ndufs4, Ndufs5, Ndufs6, Ndufs7, Ndufv1, Ndufv2, Ndufv3, Nfs1, Nme3, Nmnat3, Nubpl, Nudt19, Nudt5, Nudt7, Oaz1, Oplah, Osbpl1a, Parp16, Parp6, Pcca, Pccb, Pcyt2, Pdha1, Pdhb, Pdhx, Pdk1, Pdk2, Pdp2, Pdss2, Pecr, Pfkfb1, Pfkl, Pfkm, Pgam1, Pgd, Pgk1, Pgls, Phkg1, Pik3r1, Pkm, Plbd1, Pmvk, Pnpla2, Pnpla3, Pon1, Ppa1, Ppa2, Ppcs, Prkaa2, Prodh, Psph, Ptdss2, Ptgds, Ptges, Ptges2, Ptges3, Pts, Pycrl, Pygl, Qdpr, Rdh11, Rfk, Rida, Rpia, Sc5d, Scp2, Sdha, Sdhb, Sdhc, Sdhd, Sephs2, Shpk, Slc16a1, Slc19a1, Slc25a1, Slc25a16, Slc25a17, Slc25a20, Slc25a32, Slc37a4, Slc44a3, Slc5a6, Sord, Sqle, Sqor, Srebf1, Stard10, Stard7, Sucla2, Suclg1, Suclg2, Suox, Taldo1, Them4, Thrsp, Timmdc1, Tkfc, Tkt, Tm7sf2, Tnfaip8, Tpmt, Tspo, Tst, Ttpa, Txn1, Txn2, Uck1, Uck2, Ucp3, Ugp2, Urod, Uros, Vdac1* | 342 | 1558 | 21.95 | -8.664 | 5.29E-63 |

## Supplementary Table 4: Reactome Pathways significantly enriched in mesenteric PVAT HFD male vs chow male. Individual Reactome pathways were assessed for significant enrichment by assessing whether the number (S) of significantly differentially expressed genes within a pathway is more than expected by chance given the total number (N) of genes. Adjusted p-value (P (adj.)) was determined using a hypergeometric test and corrected for tests over multiple pathways using the Benjamini-Hochberg procedure. Z-score is calculated as $Z=(Su-Sd)/\surd N$, where Su and Sd are the number of significant upregulated and downregulated genes in the pathway respectively.

| **Reactome Pathway** | **Differentially Expressed Genes** | **S** | **N** | **S/N (%)** | **Z-score** | **P (adj.)** |
| --- | --- | --- | --- | --- | --- | --- |
| Metabolism | *Acaca, Acadsb, Acat1, Acot2, Acot4, Acsm5, Acss3, Acy1, Adhfe1, Aldh2, Aldh6a1, Aldh7a1, Alox15, Asns, Bcat2, Bphl, Car13, Car3, Car5b, Cdo1, Cept1, Ces1d, Chst1, Coq2, Crls1, Crym, Cyp2e1, Cyp2f2, Cyp2s1, D2hgdh, Dbt, Ddhd2, Echs1, Fads1, Fah, Flad1, Gcat, Gcdh, Gng4, Gpam, Gpd2, Gpt2, Gsta3, Gstk1, Gstm4, Gstm5, Gsto2, Gstt1, H2-Ke6, Hibch, Idh3b, Ivd, Kdsr, Ldhb, Maob, Mccc1, Mccc2, Mlxipl, Mmut, Mocs1, Nadk2, Nat8l, Ndufa10, Nme3, Nt5e, Osbpl1a, Pcca, Pccb, Pdha1, Pdk1, Pdk2, Pdp2, Pdpr, Pfkfb1, Phkg1, Pnp, Pnpla2, Pon1, Ppa2, Prkag2, Prodh, Ptges, Ptpn13, Pts, Rfk, Sdha, Shmt1, Slc2a3, Slc5a6, Smpd3, Sqor, Srr, Suclg2, Tkfc, Tspo, Ttpa, Uck1, Upp2, Xdh* | 99 | 1558 | 6.35 | 2.508 | 1.30E-14 |
| Branched-chain amino acid catabolism | *Acadsb, Acat1, Aldh6a1, Bcat2, Dbt, Hibch, Ivd, Mccc1, Mccc2, Shmt1* | 10 | 20 | 50 | 2.236 | 1.22E-08 |
| Metabolism of amino acids and derivatives | *Acadsb, Acat1, Aldh6a1, Aldh7a1, Asns, Bcat2, Cdo1, Crym, Dbt, Fah, Gcat, Gcdh, Gpt2, Hibch, Ivd, Mccc1, Mccc2, Nat8l, Pdha1, Prodh, Shmt1, Sqor, Srr* | 23 | 194 | 11.86 | 1.651 | 9.56E-07 |
| Pyruvate metabolism and Citric Acid (TCA) cycle | *Adhfe1, D2hgdh, Idh3b, Ldhb, Pdha1, Pdk1, Pdk2, Pdp2, Pdpr, Sdha, Suclg2* | 11 | 47 | 23.4 | 1.605 | 8.09E-06 |
| Biotin transport and metabolism | *Acaca, Mccc1, Mccc2, Pcca, Pccb* | 5 | 8 | 62.5 | 1.768 | 1.14E-04 |
| Metabolism of water-soluble vitamins and cofactors | *Acaca, Flad1, Gsto2, Mccc1, Mccc2, Mmut, Mocs1, Nadk2, Pcca, Pccb, Rfk, Slc2a3, Slc5a6* | 13 | 111 | 11.71 | 1.234 | 1.41E-03 |
| Metabolism of vitamins and cofactors | *Acaca, Coq2, Flad1, Gsto2, Mccc1, Mccc2, Mmut, Mocs1, Nadk2, Pcca, Pccb, Pts, Rfk, Slc2a3, Slc5a6, Ttpa* | 16 | 173 | 9.25 | 1.216 | 2.57E-03 |
| Regulation of pyruvate dehydrogenase (PDH) complex | *Pdha1, Pdk1, Pdk2, Pdp2, Pdpr* | 5 | 15 | 33.33 | 1.291 | 3.25E-03 |
| Pyruvate metabolism | *Ldhb, Pdha1, Pdk1, Pdk2, Pdp2, Pdpr* | 6 | 26 | 23.08 | 1.177 | 4.80E-03 |
| Glutathione conjugation | *Gsta3, Gstk1, Gstm4, Gstm5, Gsto2, Gstt1* | 6 | 27 | 22.22 | 1.155 | 5.43E-03 |
| Non-integrin membrane-ECM interactions | *Col1a2, Col3a1, Col4a1, Col4a2, Col5a2, Itga6, Pdgfa, Pdgfb, Sdc3, Vtn* | 10 | 39 | 25.64 | -1.601 | 6.18E-04 |
| Integrin cell surface interactions | *Col16a1, Col1a2, Col3a1, Col4a1, Col4a2, Col5a2, Col8a1, Comp, F11r, Icam2, Itga6, Jam3, Pecam1, Tnc, Vtn* | 15 | 69 | 21.74 | -1.806 | 4.89E-05 |
| Platelet activation, signaling and aggregation | *Abhd12, Actn1, App, Calm2, Cd9, Col1a2, Cyb5r1, Daglb, Dgkz, F2r, F2rl3, Gnai2, Gnb4, Gng11, Mapk3, Pdgfa, Pdgfb, Pecam1, Pik3r3, Pik3r6, Prkch, Psap, Rab27b, Selp, Sparc, Timp1, Vav3, Vegfc, Vegfd* | 29 | 237 | 12.24 | -1.884 | 1.28E-04 |
| Assembly of collagen fibrils and other multimeric proteins | *Col12a1, Col1a2, Col3a1, Col4a1, Col4a2, Col5a2, Col8a1, Ctsb, Ctsl, Ctss, Itga6, Lamb3, Lox, Loxl2* | 14 | 54 | 25.93 | -1.905 | 1.6E-05 |
| Degradation of the extracellular matrix | *Adam8, Adamts4, Col12a1, Col1a2, Col3a1, Col4a1, Col4a2, Col5a2, Col8a1, Ctsb, Ctsd, Ctsk, Ctsl, Ctss, Eln, Lamb3, Mmp11, Mmp12, Mmp14, Mmp15, Mmp2, Timp1* | 22 | 113 | 19.47 | -2.07 | 1.5E-06 |
| Collagen formation | *Col12a1, Col16a1, Col1a2, Col28a1, Col3a1, Col4a1, Col4a2, Col5a2, Col8a1, Ctsb, Ctsl, Ctss, Itga6, Lamb3, Lox, Loxl2, P3h1, Pcolce, Pcolce2* | 19 | 79 | 24.05 | -2.138 | 5.33E-07 |
| Hemostasis | *Abhd12, Actn1, Anxa2, App, Calm2, Capza2, Cd84, Cd9, Col1a2, Cyb5r1, Daglb, Dgkz, Dock6, Dock8, F11r, F2r, F2rl3, F7, Gnai2, Gnb4, Gng11, Gpc1, Itga6, Jam3, Kif26a, Mapk3, Nos2, P2rx5, Pde2a, Pdgfa, Pdgfb, Pecam1, Pik3r3, Pik3r6, Plau, Prcp, Prkch, Psap, Rab27b, Sdc3, Selp, Serpine2, Sparc, Sri, Tek, Timp1, Tuba1c, Vav3, Vegfc, Vegfd* | 50 | 497 | 10.06 | -2.243 | 1.65E-05 |
| Collagen degradation | *Col12a1, Col1a2, Col3a1, Col4a1, Col4a2, Col5a2, Col8a1, Ctsb, Ctsd, Ctsk, Ctsl, Mmp11, Mmp12, Mmp14, Mmp15, Mmp2* | 16 | 49 | 32.65 | -2.286 | 1.1E-07 |
| Innate Immune System | *Adam8, Aldh3b1, Anpep, Anxa2, App, Arpc1b, Arpc5, Atp6v0a1, Atp6v0d2, Atp6v1b2, C3ar1, C4b, C7, Calm2, Capza2, Casp8, Cd300lb, Cd68, Cd93, Clec12a, Crispld2, Cstb, Ctsa, Ctsb, Ctsd, Ctsk, Ctsl, Ctss, Ctsz, Dnase1l1, Dsg1a, Fabp5, Fos, Fuca2, Galns, H2-K1, Hk3, Hp, Icam2, Lcn2, Lgmn, Lilr4b, Lilrb4a, Mapk3, Mapk7, Myo10, Nck1, Nos2, Npc2, Ostf1, Pecam1, Plau, Pld3, Prcp, Psap, Ptx3, Rab31, Rab5c, Rab9b, Ripk3, S100a8, Serpinb12, Slc11a1, Tcirg1, Trem2, Tyrobp, Vat1, Vav3, Vtn* | 69 | 828 | 8.33 | -2.398 | 6.96E-05 |
| Extracellular matrix organization | *Adam12, Adam8, Adamts4, App, Bgn, Col12a1, Col16a1, Col1a2, Col28a1, Col3a1, Col4a1, Col4a2, Col5a2, Col8a1, Comp, Ctsb, Ctsd, Ctsk, Ctsl, Ctss, Eln, F11r, Fbln2, Icam2, Itga6, Jam3, Lama5, Lamb3, Lox, Loxl2, Mfap4, Mmp11, Mmp12, Mmp14, Mmp15, Mmp2, Ncan, P3h1, Pcolce, Pcolce2, Pdgfa, Pdgfb, Pecam1, Sdc3, Sparc, Timp1, Tnc, Vtn* | 48 | 248 | 19.35 | -3.048 | 5.58E-15 |

## Supplementary Table 5: Reactome Pathways significantly enriched in mesenteric PVAT HFD female vs HFD male. Individual Reactome pathways were assessed for significant enrichment by assessing whether the number (S) of significantly differentially expressed genes within a pathway is more than expected by chance given the total number (N) of genes. Adjusted p-value (P (adj.)) was determined using a hypergeometric test and corrected for tests over multiple pathways using the Benjamini-Hochberg procedure. Z-score is calculated as $Z=(Su-Sd)/\surd N$, where Su and Sd are the number of significant upregulated and downregulated genes in the pathway respectively.

| **Comparison** | **No. DEGs (Upregulated/downregulated)** |
| --- | --- |
| Chow female vs male | 61 (33/28) |
| HFD vs chow female | 590 (284/306) |
| HFD vs chow male | 773 (393/380) |
| HFD female vs male | 56 (40/16) |

Supplementary Table 6. Summary of differentially expressed genes (DEGs) in mesenteric PVAT for each comparison at adjusted P<0.05 (adjusted for multiple testing by controlling for the false discovery rate).

|  |  | **Male Chow Diet** | | | **Female Chow Diet** | | | **Male High-Fat Diet** | | | **Female High-Fat Diet** | | |
| --- | --- | --- | --- | --- | --- | --- | --- | --- | --- | --- | --- | --- | --- |
|  |  |  |  |  |  |  |  |  |  |  |  |  |  |
|  |  | **-PVAT** | **+PVAT** | ***P-*value** | **-PVAT** | **+PVAT** | ***P-*value** | **-PVAT** | **+PVAT** | ***P-*value** | **-PVAT** | **+PVAT** | ***P-*value** |
| **KPSS** | Emax (mN/mm) | 8.7 ± 0.66 | 8.1 ± 0.5 | *0.09, ns* | 8.1 ± 0.6 | 7.6 ± 0.6 | *0.09, ns* | 8.6 ± 0.7 | 8.4 ± 0.7 | *0.44, ns* | 8.4 ± 0.6 | 8.3 ± 0.6 | *0.73, ns* |
| **Phenylephrine (PE)** | EC50 [log(M)] | -5.72 ± 0.26 | -5.17 ± 0.24 | ***0.009, ***** | -6.94 ± 0.31 | -5.46 ± 0.22 | ***0.0001, ****** | -6.74 ± 0.36 | -6.12 ± 0.35 | ***0.02, **** | -6.27 ± 0.49 | -5.8 ± 0.79 | *0.09, ns* |
|  | Emax (%KPSS) | 106 ± 8.1 | 109 ± 7.6 | *0.33, ns* | 115 ± 4.1 | 111 ± 13 | *0.29, ns* | 87.9 ± 12.8 | 95.9 ± 12.3 | *0.27, ns* | 95.1 ± 17.9 | 101.6 ± 17.2 | *0.27, ns* |
| **Acetylcholine (ACh)** | IC50 [log(M)] | -7.99 ± 0.2 | -8.16 ± 0.08 | *0.13, ns* | -8.34 ± 0.27 | -8.43 ± 0.43 | *0.51, ns* | -8.1 ± 1.0 | -6.72 ± 0.94 | ***0.006, ***** | -8.1 ± 0.34 | -7.39 ± 0.8 | *0.09, ns* |
|  | Maximal relaxation (%) | 95.6 ± 6.7 | 100.7 ± 1.2 | *0.37, ns* | 99.9 ± 1.2 | 95.3 ± 9.5 | *0.37, ns* | 90.8 ± 4.2 | 73.4 ± 12.9 | ***0.016, **** | 94.5 ± 7.0 | 89.1 ± 15.3 | *0.3, ns* |
| **Sodium nitroprusside (SNP)** | IC50 [log(M)] | -8.09 ± 0.24 | -7.85 ± 0.59 | *0.51, ns* | -7.86 ± 0.61 | -7.73 ± 0.58 | *0.87, ns* | -8.09 ± 2.68 | -8.86 ± 1.66 | *0.98, ns* | -7.83 ± 0.85 | -7.28 ± 0.29 | *0.52, ns* |
|  | Maximal relaxation (%) | 91.4 ± 10.0 | 93.6 ± 11.9 | *0.97, ns* | 97.3 ±6.9 | 96.8 ± 3.3 | *0.96, ns* | 99.1 ± 0.6 | 99.5 ± 2.6 | *0.92, ns* | 100.1 ± 2.6 | 96.7 ± 3.3 | *0.33, ns* |

## Supplementary Table 7. Summary of myography data highlighting the effect of PVAT on mesenteric artery responses. Second order mesenteric arteries from male and female C57Bl6/J mice after 16 weeks of chow diet or high-fat diet. PVAT was either removed (-PVAT) or left intact (+PVAT). Data are mean ± SD (n=6-8 per group), analysed by unpaired t-test. Comparison are between -/+PVAT. *P<0.05, **P<0.01, P<0.001.


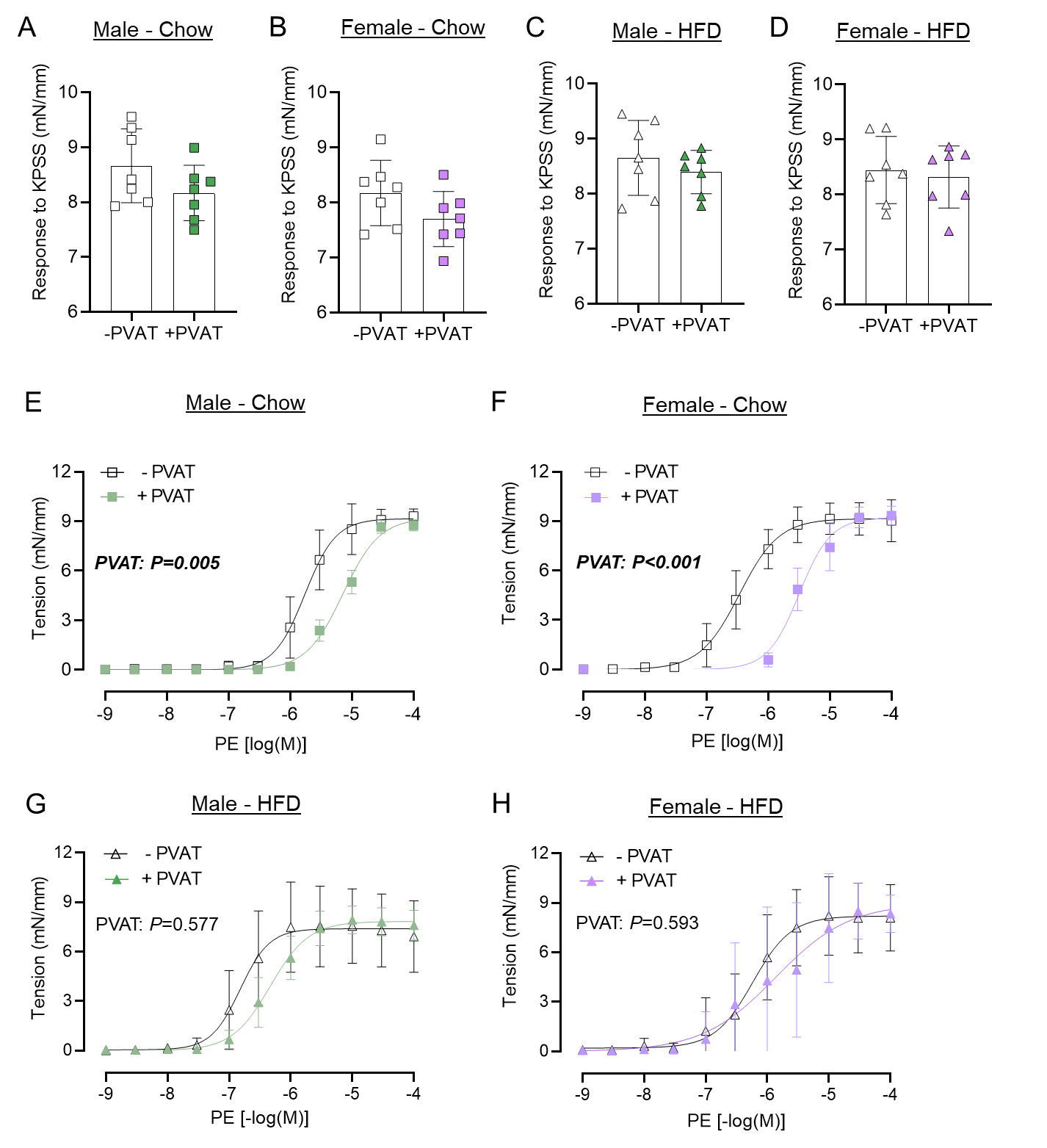


## Supplementary Figure 1. Mesenteric artery response to KPSS and phenylephrine. Second order mesenteric arteries were dissected from male (*A,C*,*E,G*) and female (*B,D*,*F,H*) C57Bl/6 mice after 16 weeks of either chow diet (Chow) or high-fat diet (HFD). PVAT was either removed (white symbols) or left intact (coloured symbols) and vessels were mounted onto a wire myograph. The response to KPSS, expressed as tension (force normalised to vessel length) was not different in any of the groups -/+ PVAT. Concentration response curves to phenylephrine (PE) were obtained (*E-H)*. For PE, contraction force was normalised to vessel length. All concentration-response curves were generated by non-linear regression. Data are mean ± SD (N=7 per group). *A-D* analysed by unpaired t-test. *E-H* analysed by repeated measures two-way ANOVA. P<0.05 was considered significant.
